# Exploring Inhibition of Bacterial Conjugation Coupling Protein TrwB: Novel Ligands to Fight Antimicrobial Resistance Spread

**DOI:** 10.1021/acsomega.5c03425

**Published:** 2025-08-01

**Authors:** Elena Gómez-Rubio, Lide Arana, Roberto Vicario-Martín, Kepa Arbé-Carton, Carlos Garbisu, Olmo Martín-Cámara, Itziar Alkorta, Sonsoles Martín-Santamaría

**Affiliations:** † Department of Molecular and Cellular Biosciences, 54446 Centro de Investigaciones Biológicas Margarita Salas, CSIC. C/Ramiro de Maeztu, 9., 28040 Madrid, Spain; ‡ Department of Applied Chemistry, Faculty of Chemistry, 16402 University of the Basque Country, C/Manuel Lardizabal, 3, 20018 Donostia, San Sebastián, Spain; § Department of Biochemistry and Molecular Biology, Faculty of Science and Technology, University of the Basque Country, Barrio Sarriena s/n, 48940 Leioa, Spain; ∥ Department of Conservation of Natural Resources, NEIKER-Basque Institute for Agricultural Research and Development, Basque Research and Technology Alliance (BRTA), Technology Park of Bizkaia, Parcela 812. C/Berreaga 1, 48160 Derio, Spain

## Abstract

Bacterial conjugation is the most sophisticated mechanism for horizontal gene transfer. Conjugative plasmids allow the recipient bacterium to acquire new traits from the donor, such as antimicrobial resistance (AMR). Among the proteins involved in the plasmid transfer machinery, the Type IV Coupling Protein (T4CP) links the relaxosome and the Type IV Secretion System (T4SS). However, despite their biological relevance and their potential as a target to control AMR, only a few T4CPs have been exhaustively studied. The archetype of the T4CP family is the coupling protein of the conjugative plasmid R388, TrwB. The inhibition of TrwB ATPase activity or oligomerization with small-molecule modulators is expected to control the transfer of R388, contributing to combat AMR spread. Following a drug repurposing approach, we have combined in silico screening studies, molecular dynamics (MD) simulations, and in vitro bacterial conjugation assays to identify a small collection of compounds that selectively decrease the frequency of conjugation of the plasmid R388 (30–40%). Our results suggest that this inhibition is the result of the specific interaction of these drugs with TrwB. The search for conjugation inhibitors, via the inactivation of proteins such as T4CPs, rises as a strategy to advance in solutions to combat the silent pandemic of AMR.

## Graphical abstract

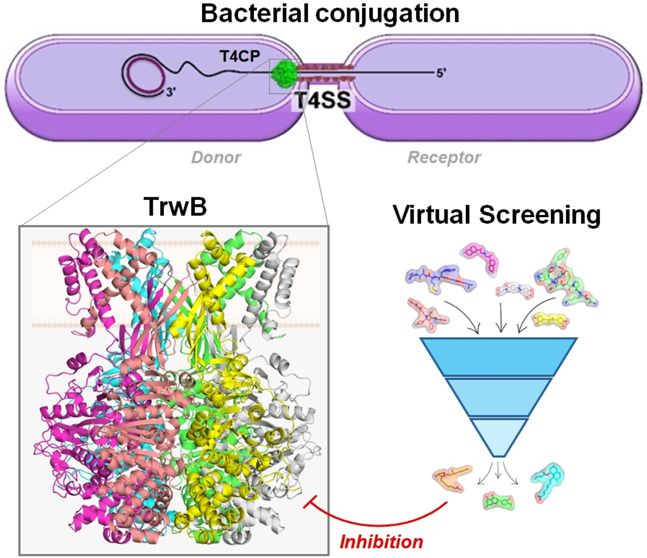

## Introduction

Apart from their role in the control of infectious bacteria, antibiotics are crucial in modern medicine, since surgery, chemotherapy, transplantation, and other medical practices greatly depend on the availability of effective antibiotics. Regrettably, their use, abuse, and misuse have caused the emergence and spread of antimicrobial-resistant bacteria, one of the topmost threats to modern medicine, becoming a leading cause of death with more than 1.2 million deaths per year.^1,2^ In order to counteract this bacterial adaptation, some new approaches have been elucidated, based on the development of new antibacterial drugs, the early identification of bacteria before antibiotic prescription, or the reduction of the transmission of antibiotic resistance genes (ARGs), among others.^3^

Antibiotic-resistant bacteria can disseminate these ARGs to other bacteria through horizontal gene transfer (HGT). One of the main mechanisms responsible for antibiotic resistance (AR) spread among bacteria is bacterial conjugation, the main HGT process in bacteria.^4^ This mechanism involves the transfer of a conjugative plasmid from a donor to a recipient bacterium. Apart from ARGs and other genes, conjugative plasmids encode all of the proteins required for plasmid transfer so that the recipient bacterium also acquires the ability to transmit the plasmid, becoming a donor (Figure 1A,B).^5^ As a consequence, the recipient bacteria acquire both AR features and the capacity to transfer the conjugative plasmid, thereby amplifying AR spread among bacteria.

**Figure 1:**
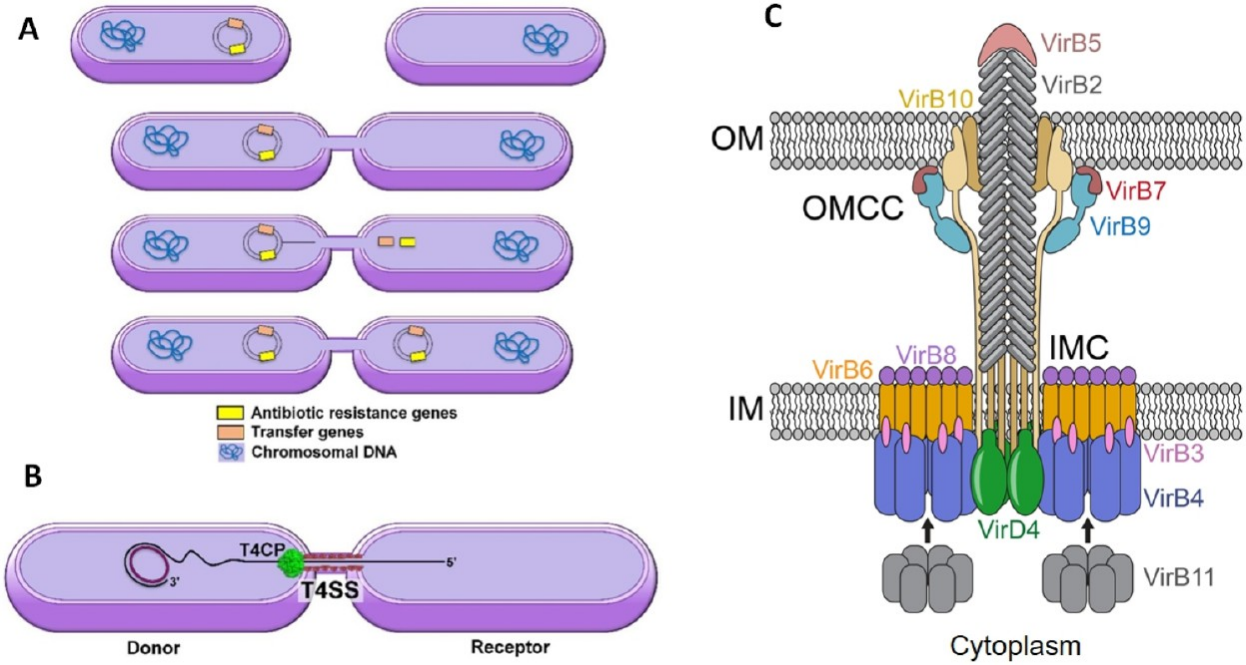
(A) Representation of horizontal gene transfer by bacterial conjugation from a donor bacterium (left) to a recipient bacterium (right). (B) Representation of plasmid transference from a donor bacterium (left) to a recipient bacterium (right) through the Type IV coupling protein (T4CP) that belongs to the Type IV secretion system (T4SS). (C) Model for the Type IV secretion system. Adapted from Alvarez-Rodriguez et al.^5^ Pilus: VirB5 and VirB2; outer membrane (OM) core complex (OMCC): the C-terminus of VirB10, VirB7, and VirB9; inner membrane complex (IMC): VirB8, VirB6, VirB3, and the N-terminus of VirB10; energy center: VirD4, VirB4, and VirB11.

The conjugative transfer is mediated by a Type IV secretion system (T4SS; Figure 1C), which is a cellular machinery capable of connecting both bacteria.^6,7^ The T4SS is a multiprotein complex consisting of a minimum of 12 different proteins. One of the key elements is the Type IV Coupling Protein (T4CP; in green in Figure 1C), which connects the plasmid to be transferred and the T4SS (Figure 1B,C).^5^ The members of the T4CP family display a high heterogeneity in sequence, length, and domain architecture, with the nucleotide-binding domain (NBD) being the only conserved domain in all T4CPs. They act as molecular engines for plasmid transfer through T4SS, driven by the energy obtained from ATP hydrolysis. Given their critical role in conjugation, T4CPs represent an interesting drug target to selectively block plasmid transfer and, hence, AR spread among bacteria.^5^

During the plasmid transfer, the action of the T4CP is responsible for the connection of the relaxosome in the cytosol and the T4SS in the membrane. The prototype protein of the T4CP family is TrwB, an ATPase that uses the energy from ATP hydrolysis for its function in *Escherichia coli* plasmid R388 conjugative transfer.^8,9^ TrwB is a 507 amino acid homohexameric membrane protein with a central pore that allows for plasmids to go in and out of the cell (Figure 2). The transmembrane domain contains the N-terminal transmembrane (TM) domains of the 6 monomers, and the cytosolic domain contains NBDs and all-α helical domains (AADs), forming an orange-shaped hexamer with a channel crossing the central part (Figure 2).^10^ So far, only the X-ray crystallographic structures of the cytosolic domain are available and have shown its resemblance to various ATPase proteins.^11^ Interestingly, it has been reported that TrwB monomers and hexamers coexist in solution when the protein is complete, but not when it lacks the transmembrane domain (soluble mutant TrwBΔN70).^12^

**Figure 2:**
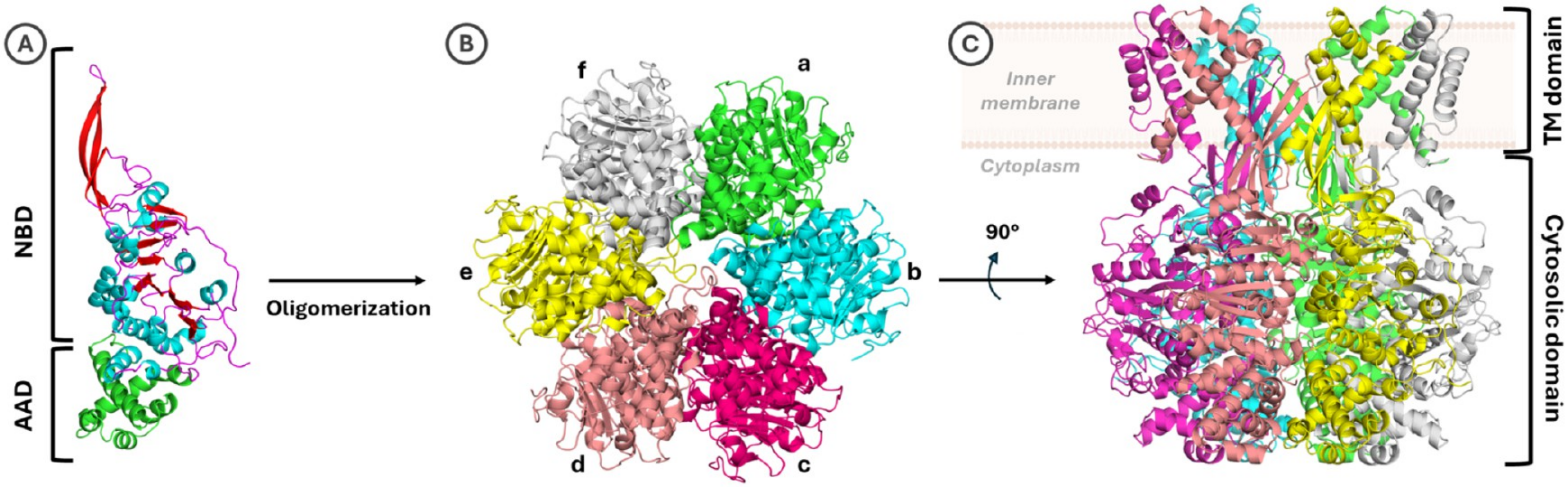
(A) Side view of the 3D structure of TrwB cytosolic subunit A from PDB 1E9S.^10^ The nucleotide-binding domain (NBD) α-helices and β-sheets are colored cyan and red, respectively. The all-α helical domains (AADs) are represented in green. (B) Top view of the 3D structure of the TrwB cytosolic domain (hexamer composed of (a–f) subunits) from PDB entry 1E9S. (C) Side view of the 3D structure from an in-house-built full-length TrwB model (unpublished results).

To control AR spread, few compounds that inhibit bacterial conjugation have already been reported^13–16^ but, in all cases, leading to nontarget effects on other bacterial processes (e.g., quorum sensing, energy metabolism, expression of regulatory genes, membrane permeability) that may affect beneficial bacterial symbionts. Therefore, a strategy targeting specific elements of conjugation, such as T4SS proteins, appears to be a safer approach.^5,17^ While research in this field has provided some modulators targeting other T4SS components, such as VirB8-like and VirB11-like proteins,^18–21^ there is currently no reported inhibitors of TrwB, one of the key DNA translocating motors of the conjugative process. We here report a multidisciplinary approach, combining in silico screening and drug repurposing studies, molecular dynamics (MD) simulations, and in vitro bacterial conjugation assays, focused on the search for small molecules that could disrupt the mechanism of action of TrwB and thus avoid bacterial conjugation and the spreading of antibiotic resistance. Our approach has led, for the first time, to the identification of TrwB-mediated conjugation inhibitors, thus opening new opportunities to understand bacterial conjugation in depth and to mitigate the spread of antimicrobial resistance (AMR).

## Results and Discussion

### Computational Search of Novel TrwB Inhibitors

Despite the pivotal role of TrwB in bacterial conjugation, research on TrwB inhibitors is scarce. Given the lack of chemical information about possible compounds that are able to bind TrwB, we decided to follow a drug repurposing approach by structure-based virtual screening of generic drug libraries. This is a well-established approach to efficiently explore the chemical diversity and to identify possible modulators of a given target with a known three-dimensional (3D) structure. We aimed to find novel small molecules able to inhibit or at least interfere with the TrwB-mediated conjugation. This approach would therefore provide us with clues about possible scaffolds with the ability to modulate TrwB for further drug design. The X-ray crystallographic structure of a soluble version of TrwB lacking the transmembrane part (TrwBΔN70)^12^ shows that the molecule consists of six equivalent protein monomers to form an almost spherical quaternary structure with a central channel, 20 Å in width, that traverses the hexamer (PDB 1E9S).^10^ Therefore, we envisaged this study by focusing on the cytosolic domain of TrwB and the following two main strategies for in silico screening: (i) to consider the TrwB hexamer as a whole, targeting the internal channel for DNA transfer, and (ii) to consider the individual TrwB monomer, targeting pockets at the dimerization interface in order to hamper the oligomerization and the subsequent functioning of TrwB.

#### TrwB Hexamer Channel: Binding Site Search and Virtual Screening

The first strategy was to target the internal channel (ICH) of the TrwB hexamer, with the aim of identifying small molecules able to block the transfer of plasmids. To perform the virtual screening, three regions along the length of the channel were defined: channel sections I, II, and III (Figure S1). These sections were delimited based on the architecture of the channel, which is wide in the cytosolic end (sections I and II; Figure S1) and narrow in the proximity to the membrane (section III; Figure S1). We also considered key residues reported:^22^ residue N271, which blocks the entrance to the ICH, conserved W216, whose mutation resulted in a protein not able to hydrolyze ATP and able to assemble with wild-type monomers, leading to inactive mixed hexamers, and K421, one of the three lysines protruding into the ICH. With these premises, we centered the grids on residues N271 for section I, W216 for section II, and K421 for section III. Besides, section I was divided into two subsections, Ia and Ib, with different grid sizes.

Additionally, we explored the exterior surface of the TrwB hexamer cytosolic domain and found putative binders targeting the intermonomer grooves to disrupt the TrwB (de)­oligomerization process. We selected the groove between chains A and B from PDB 1E9S and defined it as section IV with three subsections (IVa, IVb, and IVc), centering the grids on residues located along the interface between monomers R86, R124, and R318, respectively (Figure S1).

The WORLD collection of generic drugs from the ZINC database,^23,24^ consisting of 2492 compounds, was selected to perform computational screening with Glide (see the Materials and Methods section). The screening protocol consisted of a high-throughput virtual screening (HTVS), followed by a standard precision (SP) protocol and, finally, an extra precision (XP) protocol. Then, the top 20% of ligands with the best binding score were selected for deeper analysis (results not shown). After a combination of the score ranking, analysis of the resulting binding poses, and visual exploration of the docked complexes, four compounds that bound at hexameric TrwB were selected for further studies: nordihydroguaiaretic acid (NDGA), ZINC000000012342; nebivolol, ZINC000005844788; pravastatin, ZINC3798763; and hesperetin, ZINC000000039092 (chemical structures can be found in Figure S2). As predicted by docking calculations, these four compounds were bound to the TrwB internal channel at the cytosolic entrance (sections I and II, Figures 3 and S1). Subsequently, the resulting complexes between the TrwB hexamer and the selected compound were submitted to molecular dynamics simulations (Figure S3) in order to study their stability and to describe the TrwB–ligand interactions.

**Figure 3:**
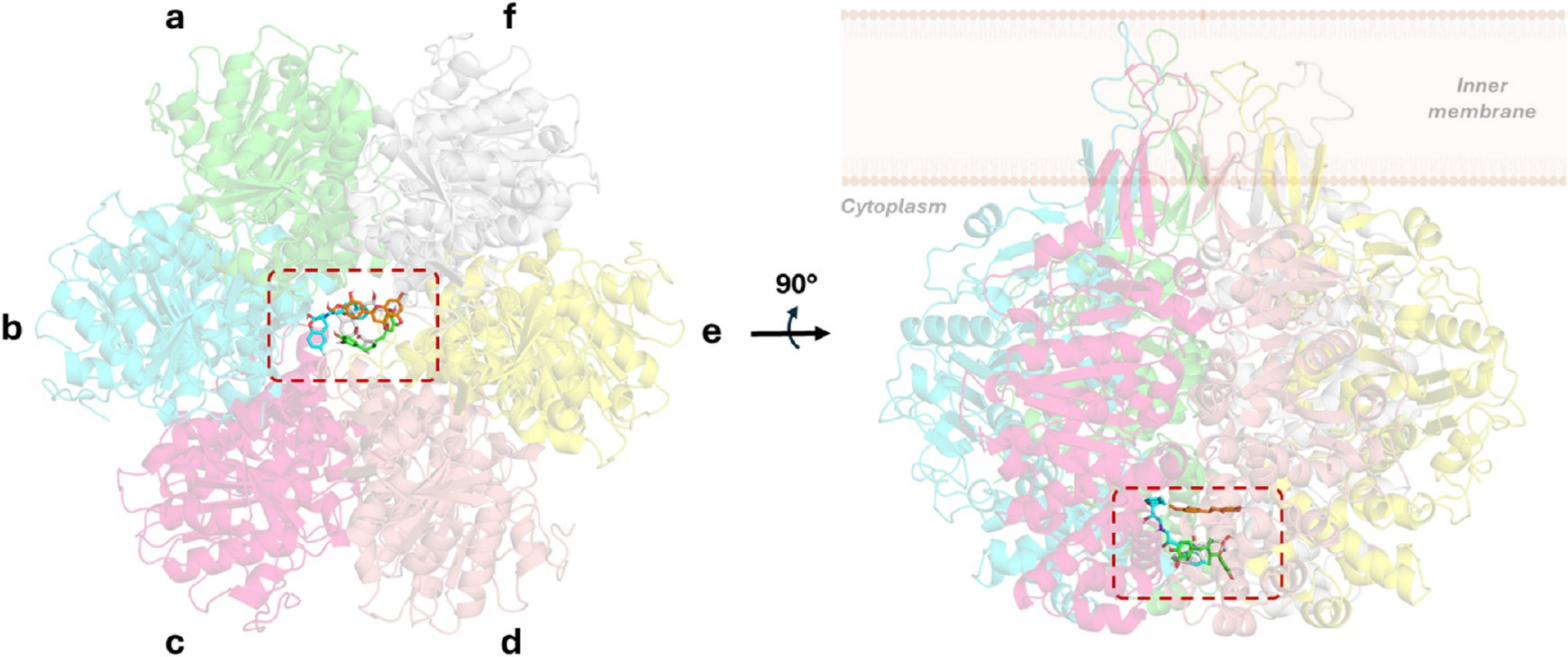
Top view (left) and side view (right) of the superimposed complexes obtained from virtual screening of TrwB with nordihydroguaiaretic acid (green), nebivolol (cyan), pravastatin (gray), and hesperetin (orange). Each subunit of TrwB (a–f) is shown in a different color, and the ligands are highlighted in a dashed red square. Nonpolar hydrogens have been omitted for clarity.

#### Nordihydroguaiaretic Acid (NDGA)

The NDGA was predicted to bind across the internal channel of the TrwB hexamer, in close contact with section I (Figure 3) mainly represented by residues E214, N271, and E272 of subunits B, C, D, E, and F and establishing hydrogen bonds between the catechol moiety and residues E272 (C and E) and N271 (E). Due to the proximity of lysins to the catechol rings, π–cation interactions are also observed with K275 from subunits C and E. Regarding the methyl groups, one of them is allocated in section I of subunit D, while the other is oriented toward the center of the channel.

During the MD simulation of the TrwB–NDGA complex, the ligand moves from the center of the channel to the inner intermonomer region between the B and C chains, between the α helices formed by residues E212-K230 and N271-T293 of subunit B and helix D210-A232 of subunit C, and explores different binding modes. From 450 ns onwards, the ligand adopts a new binding mode that holds the hydrogen bond interaction with E272 (C), although due to rotations of the catechol ring, this interaction alternates with a hydrogen bond with the side chain of Y219 (C). Moreover, the other catechol ring loses the interactions with section I of subunits E and F, accommodating itself between the side chains of polar amino acids of section II, mainly K209 (B), T210 (B), and D211 (C), and establishing a hydrogen bond with the side chain of T210 (B) (Figure 4). This binding mode remains stable until the end of the simulation (Figure S3).

**Figure 4:**
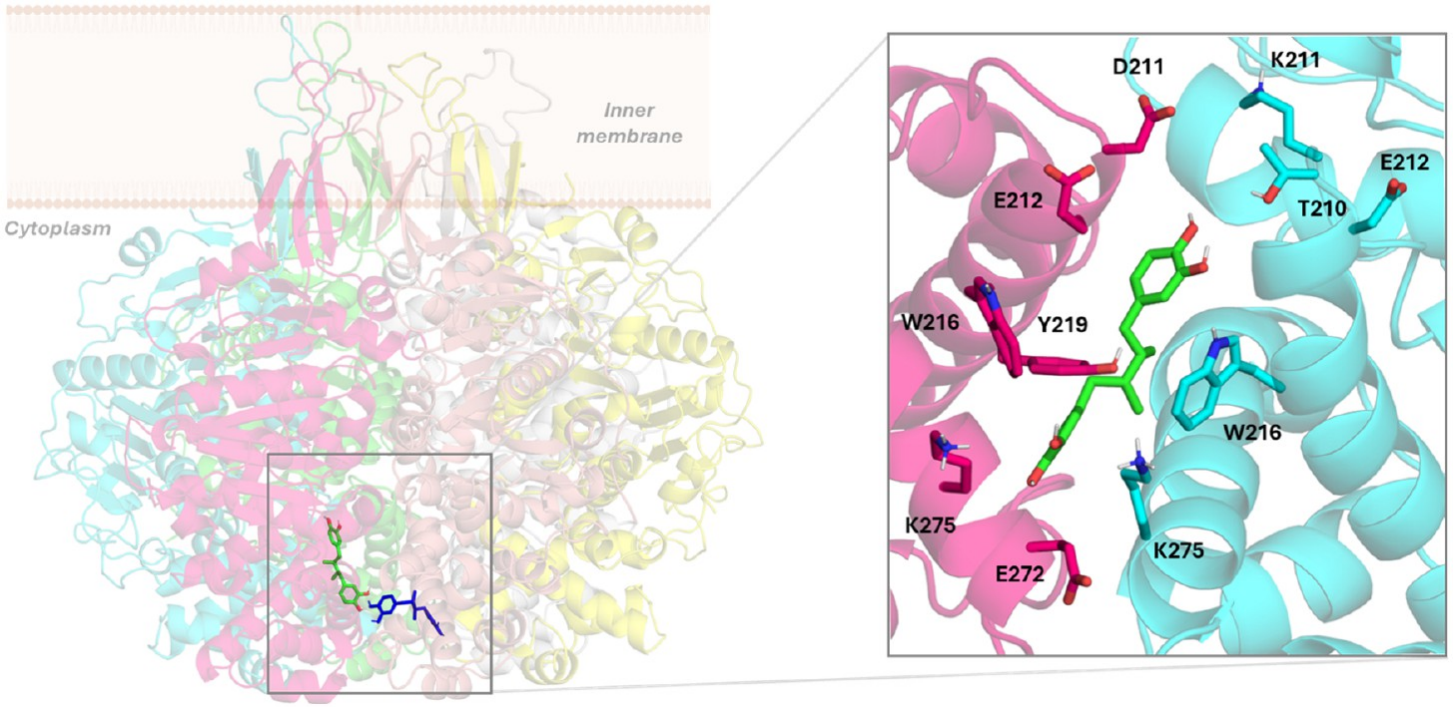
Left: superimposition of the docked pose of NDGA (blue) into the TrwB hexamer and its final binding pose (green) after 500 ns MD simulations of the TrwB–NDGA complex. Right: details of the TrwB–NDGA interactions after 500 ns MD simulations. TrwB subunits are shown in cyan (chain B) and pink (chain C). Nonpolar hydrogens have been omitted for clarity.

#### Nebivolol

Similar to the case of NDGA, nebivolol is predicted to bind at the cytosolic entrance of the TrwB channel (section I, Figures 3 and S1), where it establishes a network of hydrogen bonds and an ionic interaction, involving the quaternary amine and hydroxyl groups and the carboxylate groups from E272 from subunits B and C. The accommodation of the ligand in the channel is also favored by hydrophobic interactions with the lipophilic moieties of the side chains of residues N271 (A), N201 (B), E272 (B), N271 (C), N271 (D), and E272 (D).

When the TrwB–nebivolol complex was submitted to MD simulations, at the beginning of the trajectory, nebivolol migrates to the inner D/E interfaces within the channel, and it accommodates into the cavity formed between helices formed by residues E212-K230 and N271-T293 of subunit D and helix D210-A232 of subunit E, in a similar cavity as NDGA. Once the ligand accommodates in this position (Figure 5), at approximately 300 ns of simulation time, it remains stable until the end of the simulation (500 ns, Figure S3), establishing hydrogen bonds between the hydroxyl groups and E215 and E272 from subunit E and a π–cation interaction between the ammonium group and the W216 (D) indole ring. Furthermore, residues W216 from subunit C, W216, Y219, E212, A213, S279, and K275 from chain D, and D211, E215, and Y219 from subunit E contribute to the establishment of van der Waals and hydrophobic interactions (Figure 5).

**Figure 5:**
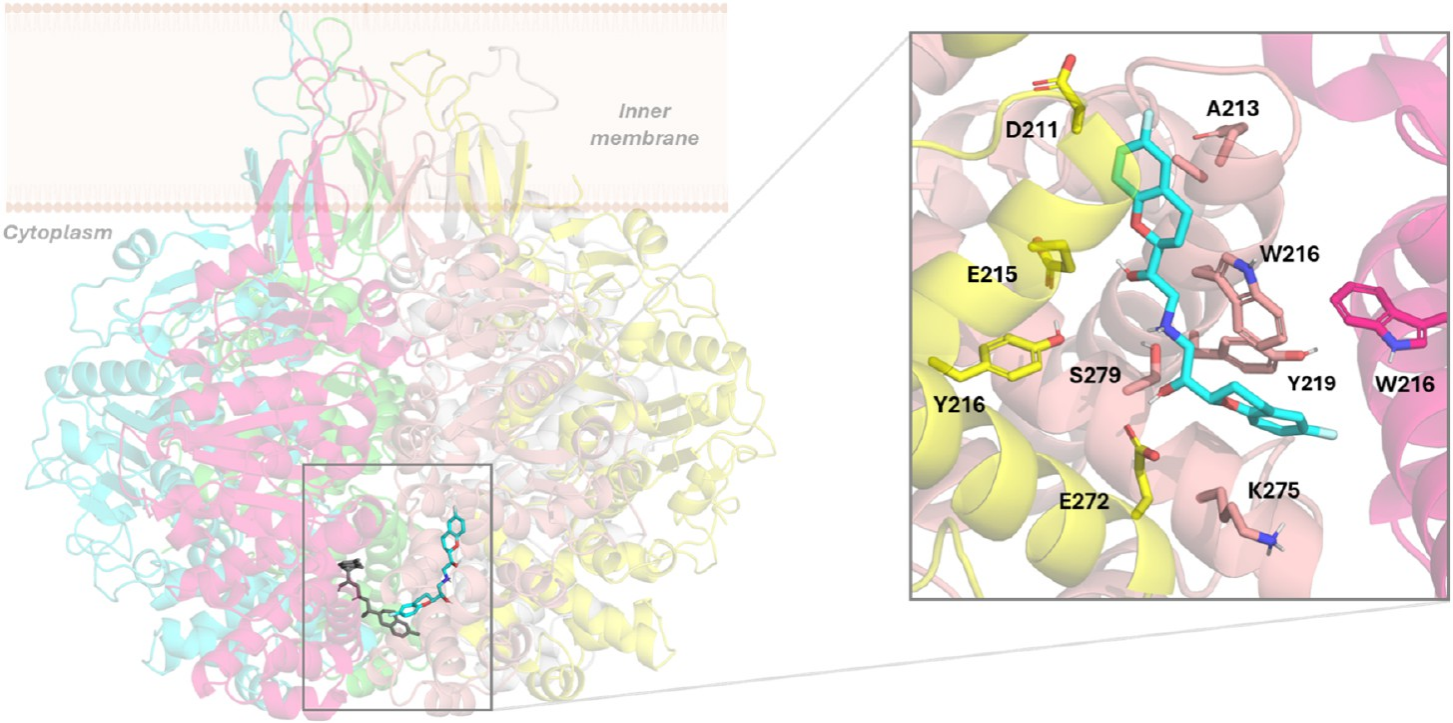
Left: superimposition of the docked pose of nebivolol (gray) into the TrwB hexamer and its final binding pose (cyan) after 500 ns MD simulations of the TrwB–nebivolol complex. Right: details of the TrwB–nebivolol interactions after 500 ns MD simulations. TrwB subunits are shown in pink (chain C), salmon (chain D), and yellow (chain E). Nonpolar hydrogens have been omitted for clarity.

#### Pravastatin

In the case of pravastatin, the docked binding pose is characterized by being located in the center of the channel in section I (Figures 3 and S1). The hydroxyl group from the bicycle and the hydroxyl groups from the aliphatic chain interact through hydrogen bonds with the N271 (C) amide side chain and E272 (F) carboxylate, respectively. Furthermore, the pravastatin carboxylate together with the K275 (E) ammonium group establishes an ionic interaction reinforced by hydrogen bonds. Finally, the aliphatic moieties from residues N271, E272, and K275 from subunit D and also N271 from subunits E and F participate in hydrophobic interactions with pravastatin.

Similar to the ligands described above, at the early stage of the simulation of the TrwB–pravastatin complex, pravastatin also migrates to a less exposed region in the channel and, interestingly, accommodates into the same groove as described for nebivolol and NDGA: the groove delimited by residues E212-K230 and N271-T293 of subunit D and helix D210-A232 of subunit E. This bound mode remains stable throughout the simulation (Figure S3) and is characterized by the pravastatin ring oriented facing the protein while the carboxylic acid is oriented toward the channel and interacts with K275 (C and D) side chains through ionic interactions reinforced by hydrogen bonds. At the opposite extreme of the ligand, the carbonyl oxygen of the ester group establishes a hydrogen bond with the OH group of S279 (D). In addition, hydrophobic interactions that also favor this binding mode involve residues E212, W216, S279, and F282 from subunit D and Y219, E272, and A276 from subunit E (Figure 6).

**Figure 6:**
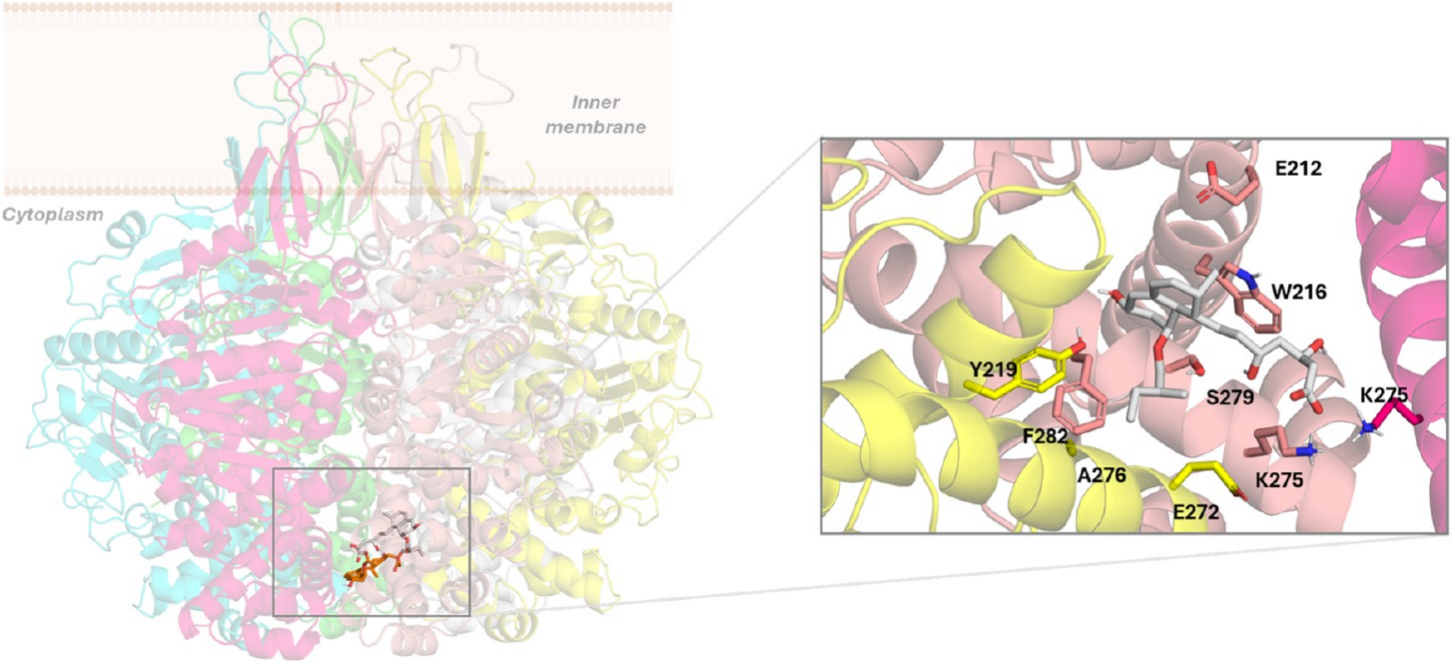
Left: superimposition of the docked pose of pravastatin (orange) into the TrwB hexamer and its final binding pose (pale gray) after 500 ns MD simulations of the TrwB–pravastatin complex. Right: details of the TrwB–pravastatin interactions after 500 ns MD simulations. TrwB subunits are shown in pink (chain C), salmon (chain D), and yellow (chain E). Nonpolar hydrogens have been omitted for clarity.

#### Hesperetin

Initially, the docked binding pose of hesperetin is similar to the docked pose for pravastatin, located at the center of the channel but, in this case, between sections I and II. Particularly, this binding mode includes a hydrogen bond between the hydroxyl group of the hydroxyanisole and E212 (hesperetin acting as a hydrogen donor) and K275 (hesperetin acting as a hydrogen acceptor), both from chain A, also between the hydroxyl group of benzopyranone and E212 (E) carboxylate, another H-bond between the carbonylic oxygen of benzopyranone and K275 (E) terminal ammonium, and a cation–π interaction through the aromatic group of hesperetin and the K275 (F) aliphatic chain.

During the first steps of the simulation of TrwB–hesperetin, the ligand moves from the starting docked pose to a new site nearby, where the molecule remains stable when (Figure S3) inserted into a cavity between subunits A and F during the simulation. This happens to be the same cavity as that described for the previous compounds (residues E212-K230 and N271-T293 of subunit F and helix D210-A232 of subunit A). In this new binding site (Figure 7), the hesperetin benzopyranone ring forms transient polar interactions between the carbonyl oxygen and the hydroxyl group of Y219 (A) and between the hydroxyl group of the hydroxyanysole moiety and the carboxylate of D211 (A). These interactions are reinforced by the CH–π interactions of pyranone and the W216 (F) side chain, the hydrogen bond between the hydroxyl group of benzopyranone and the D286 (F) carboxylate, and other nonpolar interactions between the methyl group of hydroxyanysole and the A213 (F) side chain. It is worth mentioning that other docked binding poses were simulated and, remarkably, most of them converged to the same final binding pose into the equivalent cavity at the interface of two subunits, always delimited by residues D211, A213, Y216, Y219, and E286. This convergence among the different TrwB–hesperetin complex simulations reinforces this cavity as the possible binding site for this drug. Remarkably, this convergence is also extended to NDGA, nebivolol, and pravastatin, whose final binding site is the same, indicating the strong tendency of this pocket to allocate small molecules.

**Figure 7:**
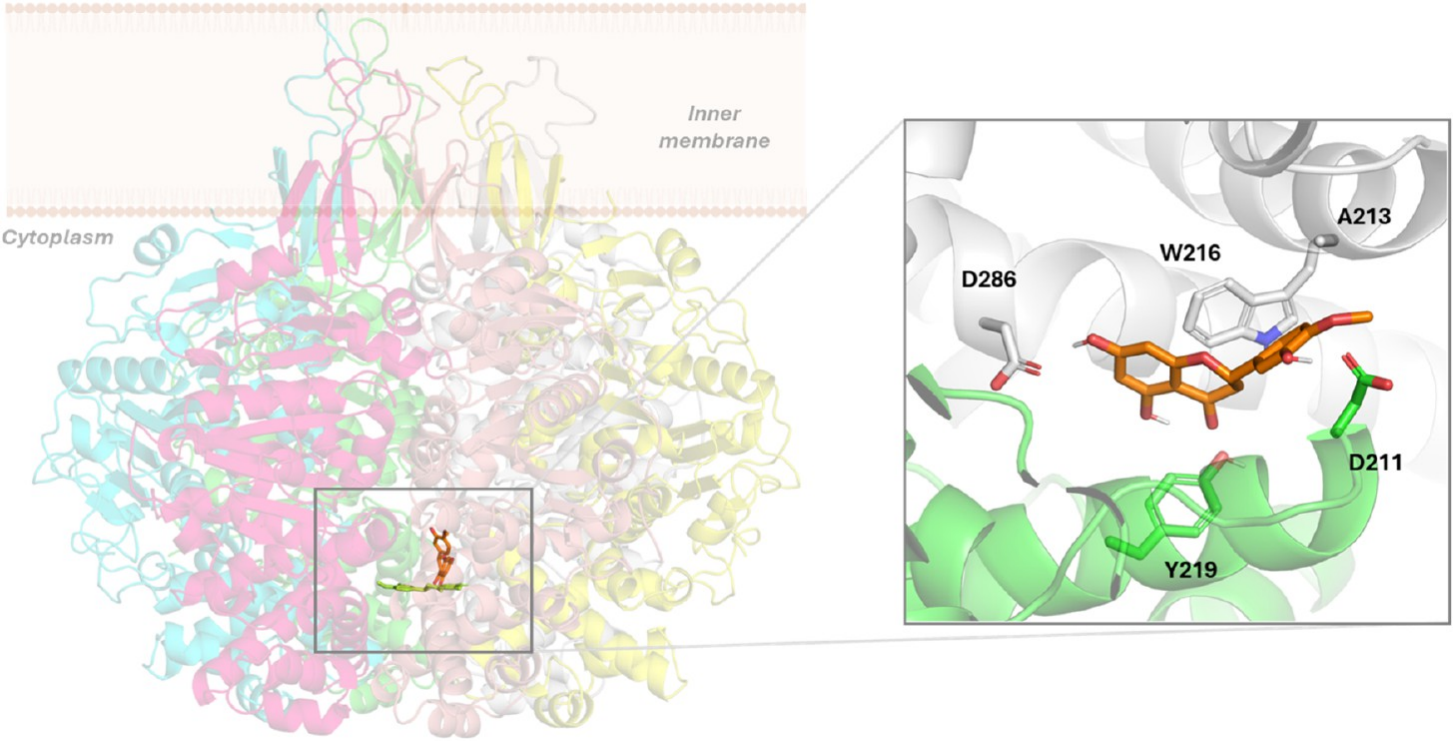
Left: superimposition of the docked pose of hesperetin (lemon green) into the TrwB hexamer and its final binding pose (orange) after 500 ns MD simulations of the TrwB–hesperetin complex. Right: details of the TrwB–hesperetin interactions after 500 ns MD simulations. TrwB subunits are shown in green (chain A) and gray (chain F). Nonpolar hydrogens have been omitted for clarity.

#### TrwB Monomer Mapping and Virtual Screening for Modulator Searching

We also explored the TrwB monomer in order to find possible binding sites whose blocking might prevent oligomerization. For the monomer, the VS was performed using chain A from the same X-ray structure of the hexamer (PDB entry 1E9S). The search was performed using SiteMap,^25,26^ and as a result, five pockets were identified (Figure 8, and details are in the Supporting Information). The first pocket (site 1) corresponded to the nucleotide-binding site; therefore, it was discarded. The second pocket (site 2) accommodates the loop formed by the amino acids V412–P420 of the adjacent monomer. The third pocket (site 3) corresponded to a region away from the oligomerization interface; therefore, it was also discarded. The fourth pocket (site 4) and the fifth pocket (site 5) are contiguous, so they were considered to be taken together for the in silico screening.

**Figure 8:**
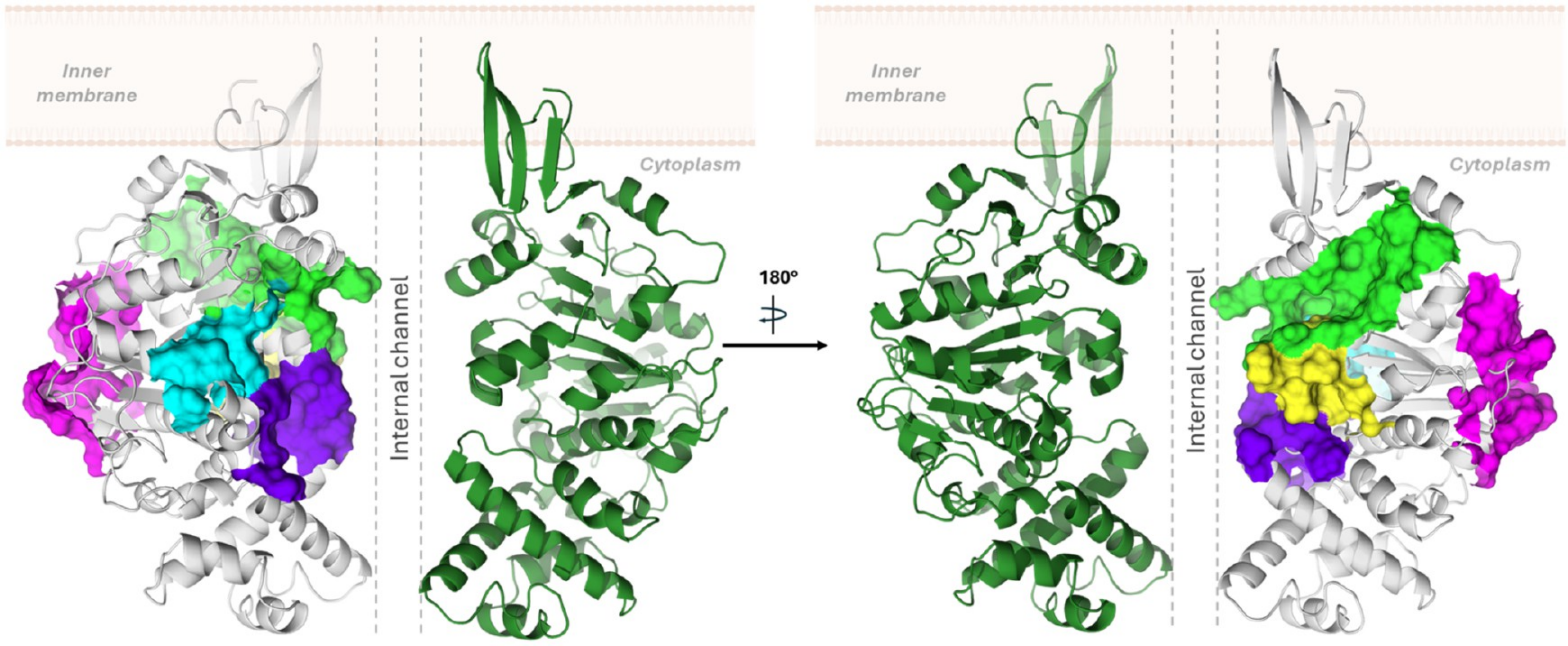
Representation of the longitudinal cut of the TrwB cytosolic domain. Subunits A, C, D, and F are not shown, and the internal channel (ICH) is marked by dashed lines. The identified binding sites are represented as the surface (site 1, cyan; site 2, green; site 3, magenta; site 4, blue; site 5, yellow).

The virtual screening was performed in sites 2, 4, and 5 (data not shown). However, due to its remarkable characteristics in terms of search for potential oligomerization disruptors, we focused on site 2 for deeper studies because it accommodates a loop of the adjacent monomer (V412–P420), and therefore, its occupation could prevent the interaction between monomers. For the VS, the same ZINC collection as in the hexamer was used (see the Materials and Methods section). After the computational calculations, five compounds were selected (atorvastatin, ZINC3920719; balsalazide, ZINC3952881; dinoprost, ZINC3830709; pentagastrin, ZINC95617677; felipressin, ZINC13307544) to further study the binding mode by performing MD simulations of the resulting complexes with the TrwB monomer. Due to the impossibility of acquiring felipressin, we searched for analogues, and after docking studies with favorable results, terlipressin (DrugBank ID DB02638) was selected to continue with the studies. All of the complexes from VS are represented in Figure 9. The chemical structure of each compound can be found in Figure S4.

**Figure 9:**
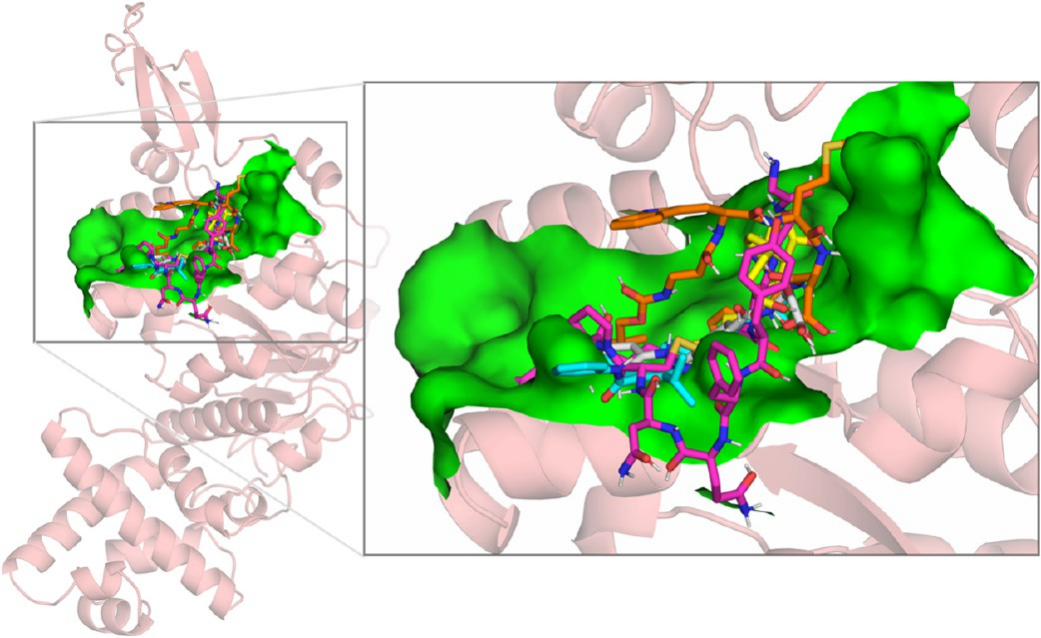
Superimposition of monomeric TrwB (salmon) in complex with terlipressin (pink), balsalazide (white), pentagastrin (orange), atorvastatin (cyan), and dinoprost (yellow) from the virtual screening. All compounds interact with site 2, depicted in green. Nonpolar hydrogens have been omitted for clarity.

#### Terlipressin

This synthetic peptide binds to site 2 of monomeric TrwB, accommodating G1, G2, and G3 between loops F479–I485 and R89-R92 of TrwB, while the side chain of K10 of the ligand is enclosed by residues L127, F407, and L410 of the protein. On the other hand, the Q6 and N7 side chains of terlipressin point to the helices V397–S406 and P420-L430 of TrwB. The main ligand–protein interactions through hydrogen bonds are between the backbone of terlipressin C3 and the side chain and the backbone of R408 and G482, the G2 of terlipressin and the backbone of G482, the K10 side chain of the ligand and the F407 backbone of TrwB, the K10 backbone of the ligand and the S428 backbone, and the terminal NH group of the ligand with the side chain of D425. To complete the binding mode, the peptide establishes hydrophobic interactions with residues R92, L127, T388, R404, L410, D425, S429, G482, and R483 from TrwB.

Along the first 200 ns of the MD simulation time, terlipressin explores a new binding mode until it finds it where the K10 of terlipressin is enclosed in the cavity formed by residues L430, R408, A480, F479, I485, R483, P484, and V61, while G1, G2, and G3 are accommodated between loops F479–I485 and R89-R92, but with higher exposure to the solvent than in the initial pose. The Q6 and N7 side chains come out of the slit formed by the helices and are reoriented to the solvent. At this point, the main hydrogen bond interactions occur between the carbonyl of G1, G2, and G3 backbones of terlipressin and the guanidinium group of R92 and the NH group of the N482 side chain. Hydrogen bonds are also established between the ammonium group of K10 (terlipressin) and the carbonyl of the backbone of L36, the carbonyl of the backbone of K10 (terlipressin) and the NH group of the backbone of G481, and the carbonyl of the backbone of proline P9 (terlipressin) and the guanidinium group of R408 (Figure 10). This binding mode remains stable (Figure S5) except for subtle changes due to oscillations in the orientation of the Q6 and N7 side chains that are exposed to the solvent.

**Figure 10:**
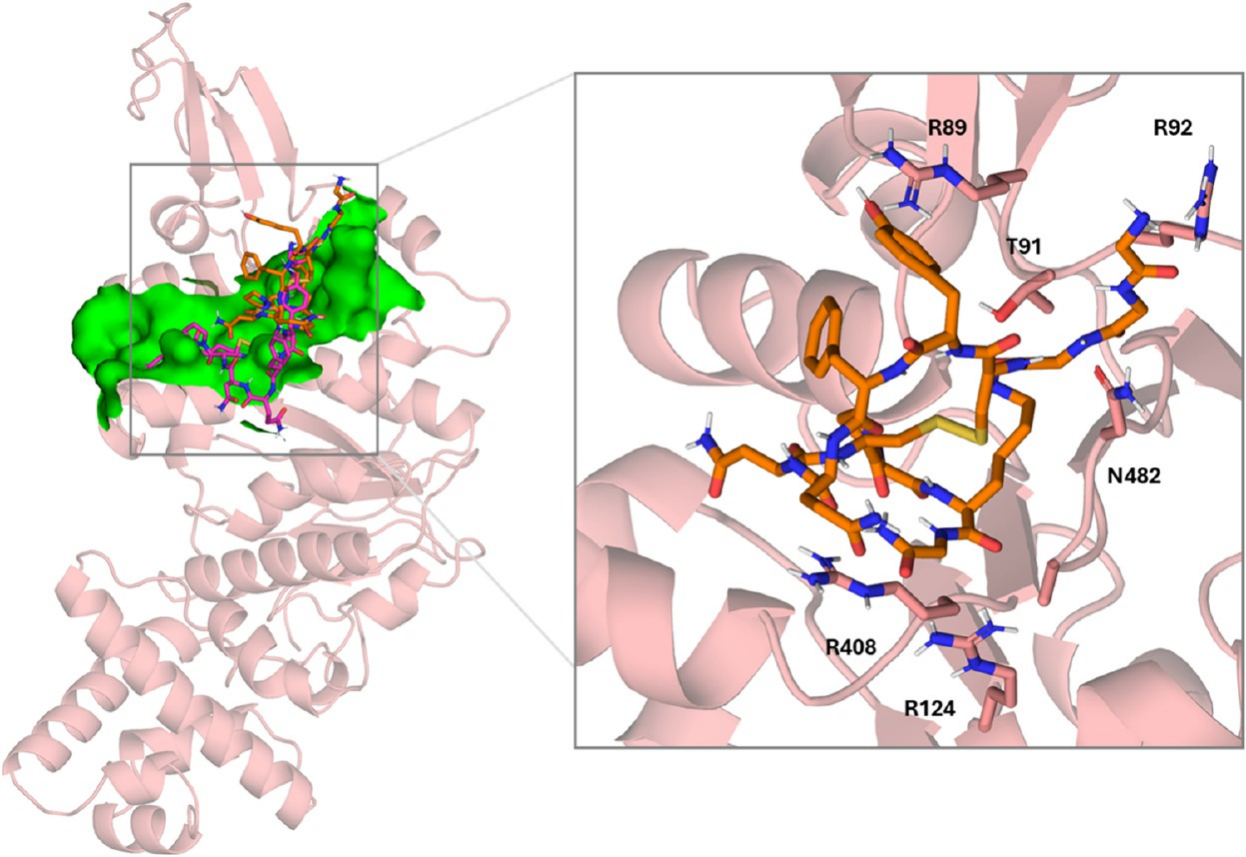
Left: superimposition of the docked pose of terlipressin (pink) into monomeric TrwB (salmon) and its final pose (orange) was achieved after 500 ns MD simulations. Site 2 is depicted as a green surface. Right: details of TrwB–terlipressin interactions after 500 ns MD simulations. Nonpolar hydrogens have been omitted for clarity.

#### Balsalazide

Balsalazide shows two ionic interactions reinforced by hydrogen bonds between the two carboxylates and the guanidinium groups of arginine residues R404 and R408. It also shows two hydrogen bonds, one between the amide group and the carbonyl of the R408 backbone and another between one of the carboxylate groups and the NH group of the G481 backbone. In addition, these same residues contribute to binding through additional hydrophobic interactions.

At the beginning of the simulation, balsalazide is displaced from site 2, where it is lodged, and for the rest of the simulation, it interacts with the surface of the V397–F407 helix. At this point, balsalazide establishes ionic interactions reinforced by the hydrogen bonds between the aliphatic carboxylate and R124 and R375 guanidinium groups and the hydrogen bonds between the amide-related carbonyl group and the guanidinium group of R124 (Figure 11). The other carboxylate group of the hydroxybenzoic moiety interacts with the NH group of the Q401 side chain via a hydrogen bond. In addition, residues T372 and A405 establish hydrophobic interactions with the hydrazineylbenzamide moiety of balsalazide, while residues Q401 and T402 establish hydrophobic interactions with the hydroxybenzoic moiety of balsalazide. Once this new binding mode is achieved, the complex remains stable along the MD simulation (Figure S5).

**Figure 11:**
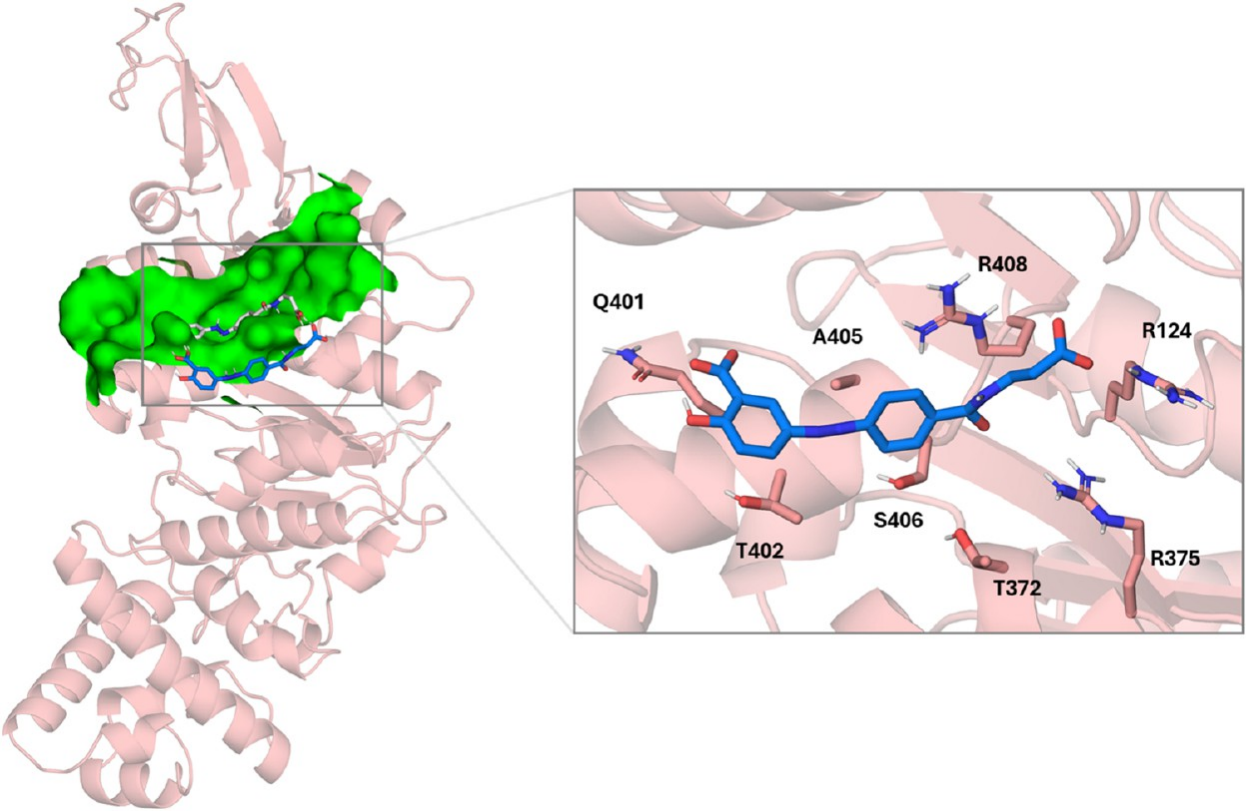
Left: superimposition of the docked pose of balsalazide (gray) into monomeric TrwB (salmon) and its final pose (blue) after 500 ns MD simulations. Site 2 is depicted as a green surface. Right: details of TrwB–balsalazide interactions after 500 ns MD simulations. Nonpolar hydrogens have been omitted for clarity.

#### Pentagastrin

This peptide establishes hydrogen bonds between the indole ring of W2 from the ligand and the E432 side chain, the carboxylate of D4 and the backbone of G481, the backbone of F5 from pentagastrin and the TrwB–F479 backbone, and the NH group and the backbone of S429. There are also ionic interactions reinforced by hydrogen bonds between the carboxylate of the ligand and the guanidinium group of R408, and hydrophobic interactions with residues G90, R92, R408, L410, L428, S429, G481, and N482.

From the beginning of the MD simulation, the ligand explores the surface of the pocket and the surrounding area. Finally, the ligand settles into a new binding mode in which the indole ring is enclosed by T91, R92, and N482, the terminal isopropyl moiety is facing the protein, establishing interactions with R89 and R458, and the methionine is exposed to the solvent. Furthermore, pentagastrin establishes an ionic bond reinforced by a hydrogen bond between the carboxylate of the D4 side chain and the guanidinium group of R92 and a π–cation interaction between F5 and the guanidinium group of R92 (Figure 12). This new binding mode is maintained along the simulation (Figure S5).

**Figure 12:**
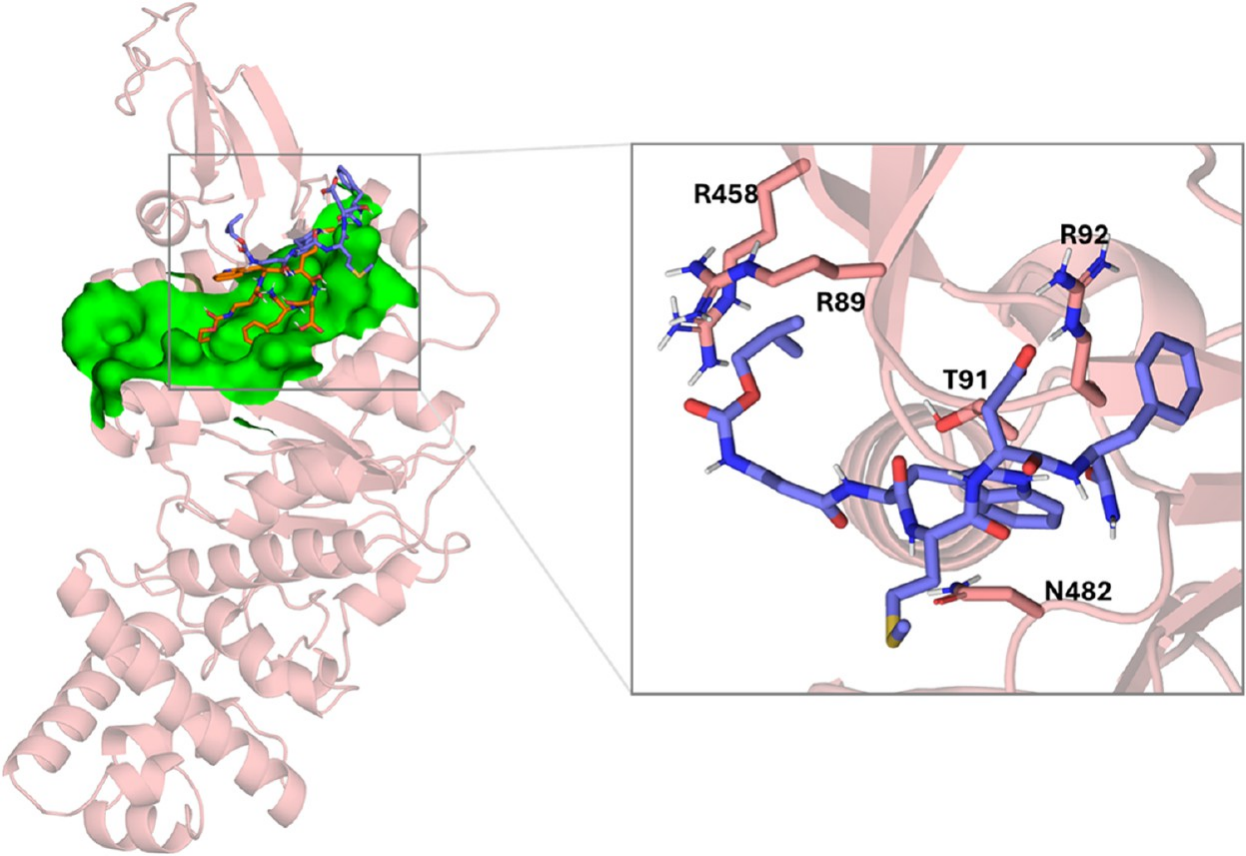
Left: superimposition of the docked pose of pentagastrin (orange) into monomeric TrwB (salmon) and its final pose (blue) occurred after 500 ns MD simulations. Site 2 is depicted as a green surface. Right: details of TrwB–pentagastrin interactions after 500 ns MD simulations. Nonpolar hydrogens have been omitted for clarity.

#### Atorvastatin

In the binding mode obtained from VS, atorvastatin binds to site 2 through hydrogen bonds between the carbonylic oxygen from the amide group and the side chain of Q401, one of the hydroxyl groups and the backbone of G481, and both hydroxyl groups and the side chain of R408. Also, there is an ionic interaction reinforced by a hydrogen bond between the carboxylate and R388 side chain. This binding pose is also favored by hydrophobic interactions with residues Q401, R404, A405, S429, and G481.

At the start of the simulation, atorvastatin slips out of site 2 to explore the surrounding surface and then returns to the same site but without being able to establish any stable binding mode. We considered that atorvastatin was not a good binder for the TrwB monomer and, therefore, discarded it.

#### Dinoprost

Dinoprost is accommodated in site 2, establishing hydrogen bonds between the hydroxyl groups from cyclopentanediol and the backbone of residue R483 and between the hydroxyl group from the octenol chain and the backbone of R408. The carboxylate group establishes an ionic interaction reinforced with a hydrogen bond with the guanidinium group of R408, which also interacts with one of the cyclic hydroxyl groups from dinoprost by hydrogen bonding. Another hydrogen bond is formed between the carboxylate group of dinoprost and the NH of the backbone of G481. The binding mode is enhanced by the hydrophobic interactions between TrwB residues T91, R408, L410, G481, and I485 and the dinoprost lipophilic regions.

During the simulation, the octenol chain of the ligand acts as an anchorage point, buried in the cavity formed by residues L127, R408, L410, and S429 (Figure 13), reinforced by a hydrogen bond between the backbone of this last serine and the hydroxyl group. During the simulation, the cyclopentanediol moiety interacts through the hydrogen bonding between one of the hydroxyl groups and the backbone of R483, while the other hydroxyl group interacts with the NH group of the R408 backbone. On the other hand, the chain with the carboxylate that was pointing to helix R119-R124 oscillates pointing to helix S416-G431 with a hinge-like movement, and the carboxylate establishes an ionic bond reinforced by a hydrogen bond with the guanidinium group of R458 (Figure 13). This complex remains stable during the MD simulation (Figure S5).

**Figure 13:**
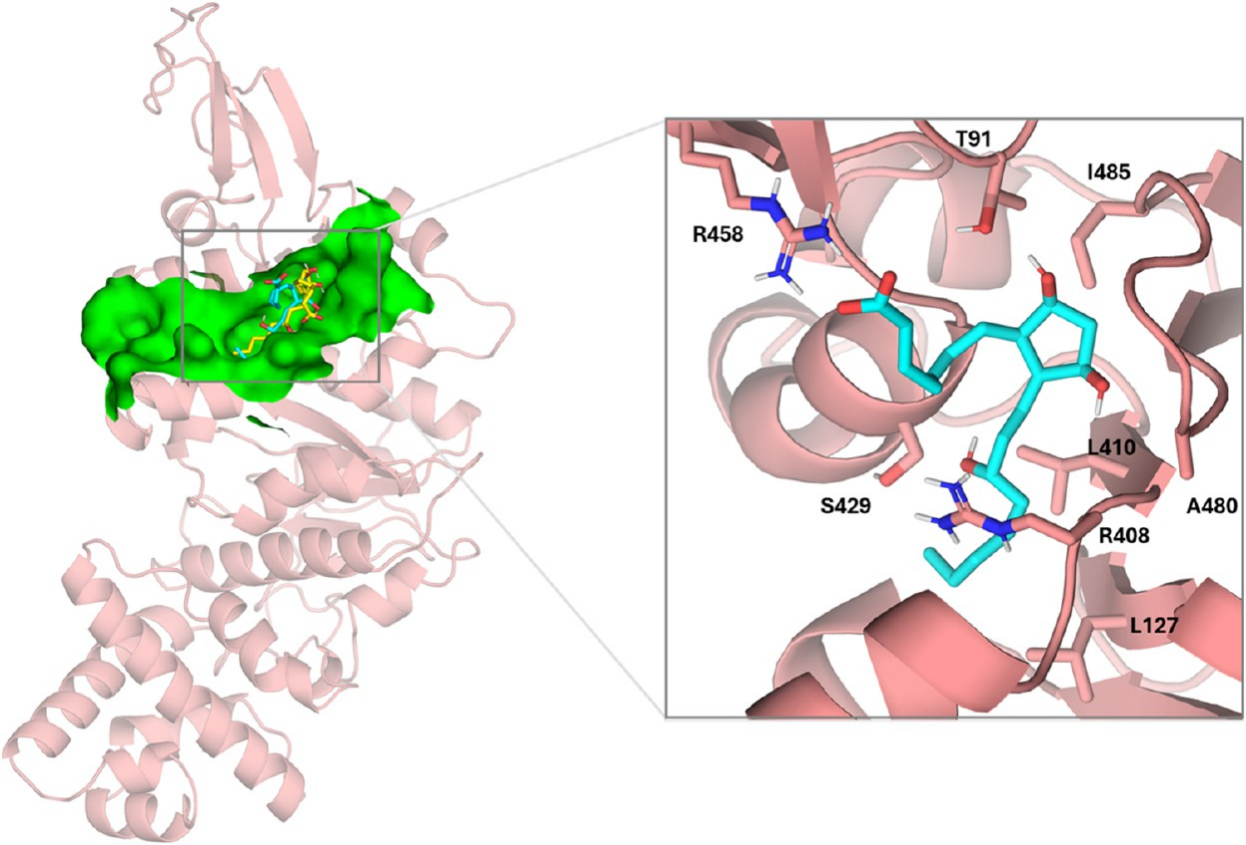
Left: superimposition of the docked pose of dinoprost (yellow) into monomeric TrwB (salmon) and its final pose (cyan) was done after 500 ns MD simulations. Site 2 is depicted as a green surface. Right: details of TrwB–dinoprost interactions after 500 ns MD simulations. Nonpolar hydrogens have been omitted for clarity.

### In Vitro Bacterial Conjugation Assays to Study the Effect of Selected Compounds on Bacterial Conjugation

A preliminary study of the effect that some of these compounds have on bacterial conjugation has been carried out (see the Materials and Methods section). For this purpose, bacterial conjugation experiments (in vitro bacterial conjugation assays) were performed in the presence of some compounds (Table 1). Nebivolol, pravastatin, and hesperetin were chosen among the compounds blocking the internal channel for DNA, and dinoprost was chosen as a compound binding site 2 of monomeric TrwB.

**Table 1:** Conjugation Frequency and Bacterial Conjugation Inhibition Percentage for Each Compound at the Tested Concentrations^a^

	mM	conjugation frequency	inhibition (%)
linoleic acid	0	2.05 × 10^–6^ ± 1.88 × 10^–7^	29.35 ± 3.28***
	0.2	1.37 × 10^–6^ ± 3.62 × 10^–8^	
nebivolol	0	1.69 × 10^–6^ ± 1.16 × 10^–7^	Ns
	0.075	1.85 × 10^–6^ ± 5.24 × 10^–7^	
pravastatin	0	4.34 × 10^–6^ ± 3.54 × 10^–7^	35.87 ± 9.40*
	1.5	2.68 × 10^–6^ ± 3.07 × 10^–7^	
hesperetin	0	2.05 × 10^–6^ ± 1.88 × 10^–7^	32.67 ± 8.60**
	2.0	1.34 × 10^–6^ ± 1.05 × 10^–7^	
dinoprost	0	2.05 × 10^–6^ ± 1.88 × 10^–7^	40.54 ± 6.05**
	2.0	1.18 × 10^–6^ ± 5.19 × 10^–8^	

a Inhibition of conjugation is expressed as a percentage relative to the control (0 mM). Data are presented as mean ± standard deviation. Statistical significance is indicated as *p < 0.05, **p < 0.01, and ***p < 0.001; ns: not significant.

To test the effect of nebivolol, pravastatin, hesperetin, and dinoprost on R388 plasmid transfer, bacterial conjugation experiments in the presence of the above-mentioned compounds were performed using a high-throughput bacterial conjugation platform.^27^ In these experiments, linoleic acid was used as a positive control of bacterial conjugation inhibition, since it has been reported to inhibit the transfer of the R388 conjugative plasmid.^28^

To rule out the possibility that the concentrations studied could affect the conjugation frequency values by inhibiting the bacterial growth of either donor or recipient strains, bacterial growth was analyzed in the presence of different concentrations of each compound. To this end, growth curves of donor and recipient bacteria were obtained in the presence of different concentrations of the compounds, and the maximum concentration that inhibits bacterial growth by less than 10% of the control (growth without compound) was calculated (Figures S6 and S7, donor and recipient, respectively). The results indicate the maximum concentration of each compound that limits donor and recipient growth by less than 10% (i.e., the lower of the two values). These concentrations are 0.1 mM for nebivolol, 2.2 mM for pravastatin, 2.3 mM for hesperetin, and 2.6 mM for dinoprost (Figures S6 and S7, and Table S1). The same test was performed with linoleic acid, and it was determined that the maximum concentration that could be used while inhibiting less than 10% donor and recipient growth was 2.8 mM.

To ensure that bacterial viability is not affected and that any reduction in conjugation is not due to the adverse effects of the compounds, concentrations below the established limit were used. This approach allows us to evaluate their inhibitory effect at the optimal concentrations of inhibitors for conjugation. Then, bacterial conjugation experiments were carried out in the presence of nebivolol (0.075 mM), pravastatin (1.5 mM), hesperetin (2.0 mM), or dinoprost (2.0 mM; Table S1). An inhibitory effect of pravastatin, hesperetin, and dinoprost was observed on the conjugation frequency of plasmid R388. Nebivolol did not show significant inhibition. Conversely, pravastatin (1.5 mM), hesperetin (2.0 mM), and dinoprost (2.0 mM) showed inhibition of 35.87 ± 9.40*, 32.67 ± 8.60**, and 40.54 ± 6.05**, respectively (Table 1). Linoleic acid was used as a positive control of inhibition, previously reported as a bacterial conjugation inhibitor,^28^ and in the presence of linoleic acid (0.2 mM), an inhibition of 29.35 ± 3.28*** was observed (Figure 14 and Table 1). These inhibition levels are comparable to those reported by Casu et al.^18^ (targeting the conjugative VirB8-like protein TraE after ligand optimization) and Jia et al.^15^ and Sun et al.^16^ (where adjustments of the bacterial conjugation assay enhanced inhibitory effects). Therefore, our results point to encouraging opportunities for ligand optimization, search for novel chemical entities capable of targeting the identified sites, optimization of the in vitro conjugation assay conditions, and/or improvement of intracellular delivery.

**Figure 14:**
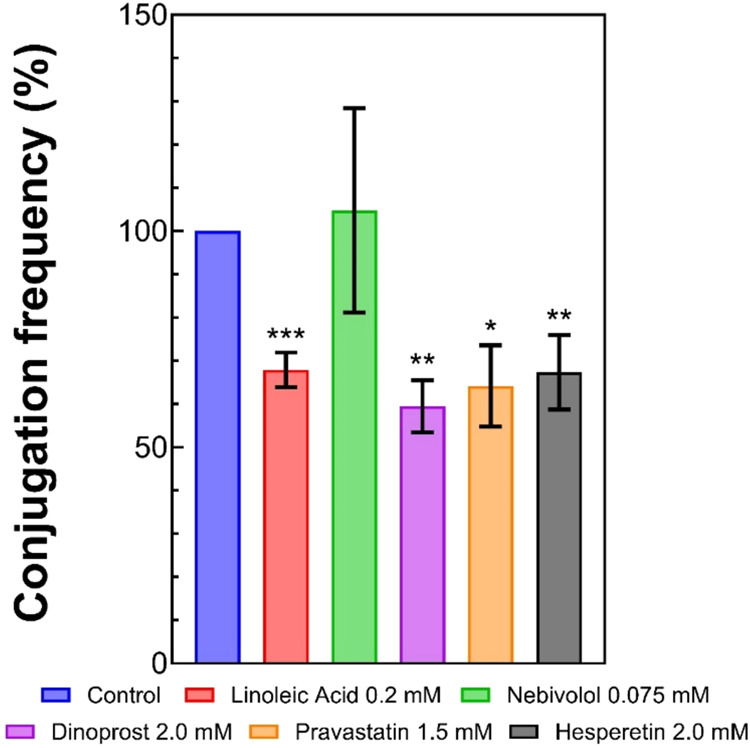
Bacterial conjugation frequency in the presence of nebivolol, pravastatin, hesperetin, and dinoprost. Data are expressed as the percentage of conjugation relative to the control value (100%, no compound). Results are the mean ± SE of at least four independent experiments performed with five conjugation replicates. The statistical significance of the observed differences with respect to the control (no compound) was analyzed by Student’s *t*-test (*p < 0.05, **p < 0.01, ***p < 0.001).

Additionally, preliminary studies with the pKM101 conjugative plasmid showed no inhibitory effect of any of the compounds studied (i.e., nebivolol, pravastatin, hesperetin, and dinoprost), suggesting that specific interaction between these ligands and TrwB could occur. All in all, the drugs here identified represent promising scaffolds for the design of novel compounds to control antibiotic resistance spread by controlling bacterial conjugation. Further cellular, biochemical, and biophysical studies are needed to fully characterize the inhibition mechanism.

## Conclusions

As a novel strategy to reduce the global health threat of antibiotic resistance spread, in this work, we have addressed the search for small molecules to inhibit the bacterial conjugation mechanism. We have used virtual screening, following a drug repurposing approach combined with MD simulations to find compounds that could bind to the monomeric or hexameric forms of the bacterial TrwB protein, thus blocking one of the key proteins of the bacterial conjugation machinery. Our results point to the cavity delimited by residues E212-K230 and N271-T293 of one subunit and helix D210-A232 of the vicinal subunit at the intermonomer groove of TrwB as a proper druggable site to find binders. Our computational protocol has allowed the identification of compounds with promising properties, among which dinoprost, pravastatin, and hesperetin have shown inhibition of bacterial conjugation in in vitro bacterial conjugation studies. These results are highly promising as they demonstrate that these compounds effectively inhibit the conjugation of conjugative plasmid R388. Our findings open up opportunities for the modulation of the transfer of other conjugative plasmids and for the elucidation of the precise mechanism of inhibition. This discovery represents a significant advancement in the development of novel strategies to hinder horizontal gene transfer in bacteria, offering potential applications for mitigating the spread of antimicrobial resistance.

## Materials and Methods

### Ligand Preparation

The substance subset WORLD, which stands for approved drugs in major jurisdictions, including the FDA, was sourced from the ZINC15 database (https://zinc15.docking.org/).^24^ Compounds that could affect the viability of the bacteria, such as antibiotics, were discarded. Subsequently, ligands underwent refinement using the LigPrep module within the Maestro Suite.^29^ This refinement process involved geometry optimization utilizing the OPLS2005 force field, determination of protonation states within a pH range of 6.0–8.0, generation of tautomers, and calculation of charges. The resultant refined library comprised 2949 ligands.

### Protein Preparation

Coordinates for the hexameric TrwB protein were obtained from its crystallographic structure (PDB 1E9S), with the monomeric structure of TrwB extracted from the A chain of the same PDB. The structures were subjected to refinement using the PrepWizard module within the Maestro Suite.^30,31^ Refinement steps included removal of water molecules and cocrystallization factors, completion of missing residues based on the FASTA sequence, addition of hydrogen atoms, determination of protonation states at pH, capping of termini, and energy minimization employing the OPSL3 force field. Specific protonation states were assigned to histidine residues, including HIE 55, HID 174, HIE 221, HIE 363, HID 377, and HIE 378.

### Pocket Search

Identification of potential binding sites was conducted through a pocket search utilizing SiteMap.^25,26,32^ The SiteMap algorithm helps identify binding sites, including allosteric binding sites and protein–protein interfaces, and evaluate their druggability. The selection criteria required at least 15 site points per reported site, and the most restrictive hydrophobicity definition was employed. A grid was subsequently generated with a cutoff set at 4 Å from the nearest site point.

### In Silico Screening

In silico screening was carried out using Glide software,^33–35^ following a hierarchical workflow.^36^ For the TrwB hexamer, given the large width and length of the inner channel and assuming there were no significant open/closed conformations, the refined crystallographic structure was used as the optimal starting structure for the screening. First, high-throughput virtual screening (HTVS) was performed, followed by standard precision (SP) and extra precision (XP) docking. In the first screening step, HTVS, the ligands were docked using the confgen method (flexible docking), and a post-docking minimization was performed. After the HTVS, the best 50% ligands were continued to be used as input for SP docking. After this step, the ligands were also minimized, and 50% of the best compounds were kept. The resulting ligand selection from the SP docking was used as an XP docking input. After the XP docking, the ligands were minimized again, and 25% of the best compounds were kept. Finally, the preliminary results from the XP docking were analyzed. For the computational screening on the TrwB monomer, the same protocol was applied, keeping 50% at HTVS, 30% at SP, and 30% at the XP stage.

### Molecular Dynamics Simulation of TrwBΔN70 and Its Complexes

Molecular dynamics simulations were performed using explicit TIP3P water arranged in a truncated octahedron box with a 10 Å distance from the protein surface. Neutralization of charges was achieved by adding Na^+^ or Cl^–^ ions. The simulations were conducted in AMBER16,^37^ utilizing the ff14SB force field for proteins and the GAFF force field for drug-like compounds. The ff14SB/TIP3P/GAFF combination was applied as it has been shown to be an optimal choice for protein/drug complexes simulations.^38^ The protocol encompassed eight stages, including minimization, heating, and equilibration. Initially, minimization involved applying harmonic potential restrictions to the solute with gradually decreasing restrictions in subsequent stages. Heating from 0 to 100 K and 100 to 300 K was carried out in the canonical ensemble using a Langevin thermostat, with restraints applied to the solute. Equilibration involved a 100 ps simulation. The production phase was extended for 500 ns under isobaric–isothermal conditions. Trajectory analysis was performed using *cpptraj* and VMD.^39,40^ The simulation protocol incorporated periodic boundary conditions, the SHAKE algorithm for hydrogen-containing bonds, and the particle mesh Ewald method to account for long-range electrostatic interactions. The Langevin thermostat was set at a collision frequency of 1.0 ps^–1^. Root-mean-square deviation (RMSD) values of the protein (Cα) and ligands (heavy atoms) were calculated for each complex during all of the simulations (Supporting Information).

### 3D Structures: Visualization, Analysis, and Figure Preparation

PyMOL^41^ was used to visualize and analyze the 3D structures of proteins and ligands and to generate the figures.

### Strains and Culture Media

*Escherichia coli* DH5α strain harboring the conjugative plasmid R388 that confers resistance to trimethoprim (TMP, 10 μg mL^–1^) and *E. coli* HMS174 strain with intrinsic resistance to rifampicin (RIF, 50 μg mL^–1^) were used as the donor and recipient, respectively, in the bacterial conjugation assays.

Lysogeny Broth (LB) culture medium^42^ was supplemented with 10 μg mL^–1^ TMP (Merck, Darmstadt, Germany) to grow donor bacteria, and recipient bacteria were grown in LB medium supplemented with 50 μg RIF mL^–1^ (PanReac AppliChem).

### Assessment of the Effect of Selected Compounds in Cell Growth

For the proper analysis of the effect of the selected compounds on the conjugation frequency, it is important to discard any alterations in the cell growth process. Therefore, *E. coli* DH5α and *E. coli* HMS174 strains were incubated in the presence of different concentrations of linoleic acid, nebivolol, pravastatin, hesperetin, or dinoprost. Since these compounds were resuspended in DMSO [0.5% (v/v)], the effect of DMSO on bacterial growth was also studied. From the results shown in Figures S6 and S7, it was observed that below 4.7% (v/v), DMSO inhibited the bacterial growth of the donor and recipient by less than 10%. Cell growth was monitored by recording OD_600_ readings every 10 min during 48 h to obtain cell growth curves for donor and recipient strains. Growth rate (μ) values for each concentration of each compound were obtained using the formula μ = (ln *N*_2_ – ln *N*_1_)/(*t*_2_ – *t*_1_), where *N*_1_ and *N*_2_ are the bacterial populations at times *t*_1_ and *t*_2_, respectively. For calculation, the *QurvE* (Quantitative growth curve Evaluation) R package was used.^43^

### Bacterial Conjugation Assay in the Presence of Different Concentrations of the Studied Compounds

For the bacterial conjugation assays, a high-throughput bacterial conjugation platform was used.^27^ Briefly, donor and recipient bacteria were grown overnight in 3.5 mL of LB supplemented with the corresponding antibiotic at 37 °C and orbital shaking at 220 rpm. Cultures were then diluted with LB to an OD_600_ of 0.6. Next, 100 μL of the donor culture was mixed with 100 μL of the recipient culture. The mixture was centrifuged at 6000*g* for 4 min, and the pellet was resuspended in 50 μL of LB and added to the well. After applying vacuum and fixing the cells to the filter, 150 μL of LB supplemented with different concentrations of nebivolol (0.075 mM), pravastatin (1.5 mM), hesperetin (2.0 mM), or dinoprost (2.0 mM) were added to the well. Then, the plate was incubated at 37 °C for 5 h on an LB agar circular plate. Linoleic acid was used as a positive inhibition control at the following concentrations: 0, 0.1, and 0.2 mM. Next, 150 μL of each well was removed by vacuum filtration, and cells were detached from the filter by adding 200 μL of 0.8% (w/v) NaCl, followed by pipet mixing 10 times. Subsequently, 10 μL of this suspension was inoculated into a sterile 96-well flat-bottomed microplate (TC-Plate 96 Well, Standard, R, Sarstedt, Nümbrecht, Germany), and 140 μL of LB supplemented with RIF for the selection of recipients and RIF and TMP for the selection of transconjugants was added. Plates were incubated at 37 °C for 24 h with shaking (218.3 rpm) in an Infinite MNano plate reader (Tecan, Männedorf, Switzerland), and OD_600_ readings were taken every 10 min to obtain the growth curves for recipients and transconjugants. The time needed to reach the threshold value (OD_600_ = 0.3) in the growth curves, i.e., “time to threshold,” was used to calculate the number of recipients and transconjugants as described previously.^27^ Plasmid transfer frequency was determined as the ratio of the number of transconjugants per milliliter to the number of recipients per milliliter (CFU_T_ mL^–1^/CFU_R_ mL^–1^). All assays were performed with at least four independent experiments, each with five conjugation replicates and three technical replicates. The corresponding conjugation assay controls based on the expected antibiotic resistance phenotypes were carried out both for the calculation of donors and recipients and to rule out contamination of the cultures.

### Graphical Representations and Statistical Analysis

Bacterial growth rates (Figures S6 and S7) were analyzed using *QurvE* and then graphed using GraphPad Prism 10 (GraphPad Software, San Diego, CA). The percentage of conjugation frequencies (Figure 14) was also graphed using GraphPad Prism 10 (GraphPad Software, San Diego, CA). Statistical analysis carried out to search for statistically significant differences in plasmid transfer frequencies was performed using two-tailed unpaired Student’s *t*-test using GraphPad Prism 10 (GraphPad Software), where *p* < 0.05 was considered to be significant.

## Supplementary Material



## Notes

Representation of the sections defined for virtual screening in the TrwB cytosolic domain; chemical structures of the selected compounds from the virtual screening; RMSD plots for the MD simulations; pockets identified with SiteMap in the TrwB monomer; effect of DMSO, linoleic acid, nebivolol, pravastatin, hesperetin, and dinoprost on donor and recipient bacterial growth; and additional data of the bacterial conjugation inhibition experiments (PDF)
