# Supplementary material for: Exploring Inhibition of Bacterial Conjugation Coupling Protein TrwB: Novel Ligands to Fight Antimicrobial Resistance Spread
Source: ACS Omega. 2025 Aug 1;10(31):34645–58. doi: 10.1021/acsomega.5c03425 (PMC12355425; doi:10.1021/acsomega.5c03425)
Supplement: Supplementary file 1 [file ao5c03425_si_001.pdf]

Supporting Information

Exploring inhibition of bacterial conjugation coupling protein TrwB: novel ligands to fight antimicrobial resistance spread

Elena Gómez-Rubio[1], Lide Arana[1,2], Roberto Vicario-Martín[1], Kepa Arbé-Carton[3], Carlos Garbisu[4], Olmo Martín-Cámara[1], Itziar Alkorta[3], Sonsoles Martín-Santamaría[1*]

[1]Department of Molecular and Cellular Biosciences, Centro de Investigaciones Biológicas Margarita Salas, CSIC. C/ Ramiro de Maeztu, 9. 28040-Madrid, Spain.
[2]Department of Applied Chemistry, Faculty of Chemistry, University of the Basque Country. C/ Manuel Lardizabal, 3. 20018-Donostia-San Sebastián, Spain.
[3]Department of Biochemistry and Molecular Biology, Faculty of Science and Technology, University of the Basque Country. Barrio Sarriena s/n. 48940-Leioa, Spain.
[4]Department of Conservation of Natural Resources, NEIKER-Basque Institute for Agricultural Research and Development, Basque Research and Technology Alliance (BRTA). Technology Park of Bizkaia, Parcela 812. C/ Berreaga 1. 48160-Derio, Spain.

*Corresponding author. E-mail: smsantamaria@cib.csic.es

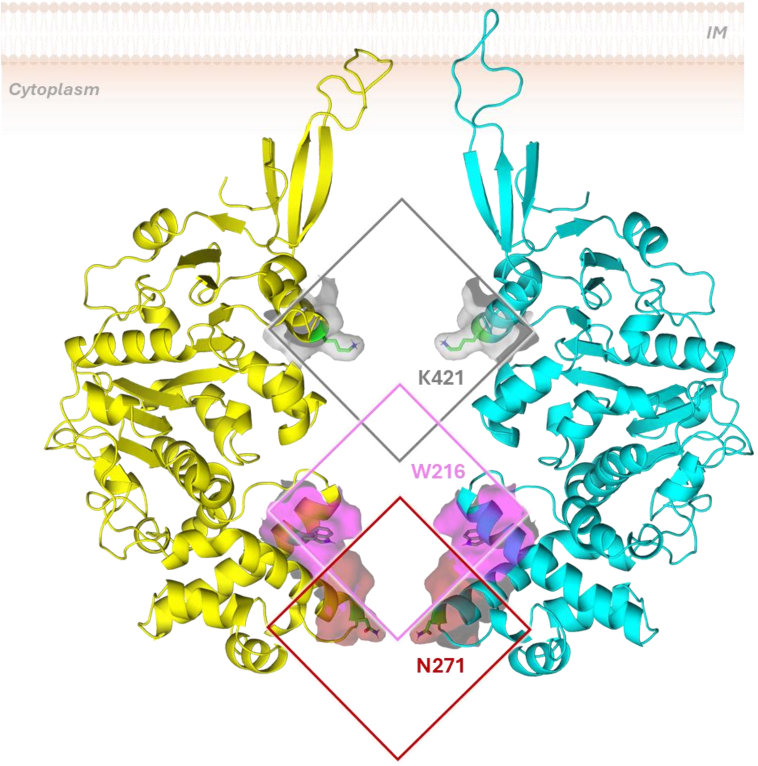

Figure S1. Representation of the longitudinal cut of TrwB cytosolic domain. Subunits A, C, D and F are not shown. The three main sections defined for virtual screening are highlighted in squares and the surface of the binding area is represented as a surface (red for section I, pink for section II and grey for section III). The residues used for centering each grid are represented in sticks.

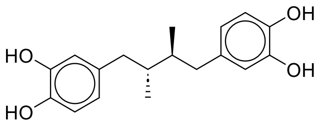

ZINC000000012342
Nordihydroguaiaretic acid
(NDGA)

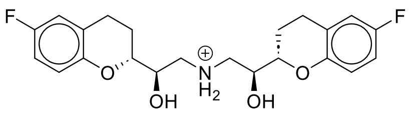

ZINC000005844788
Nebivolol

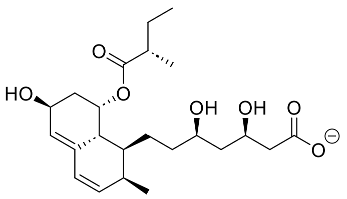

ZINC3798763
Pravastatin

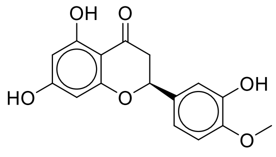

ZINC000000039092
Hesperetin

Figure S2. Chemical structure of selected compounds from the virtual screening in the hexamer. The protonation states are depicted as they were employed for the VS and the MD simulations.

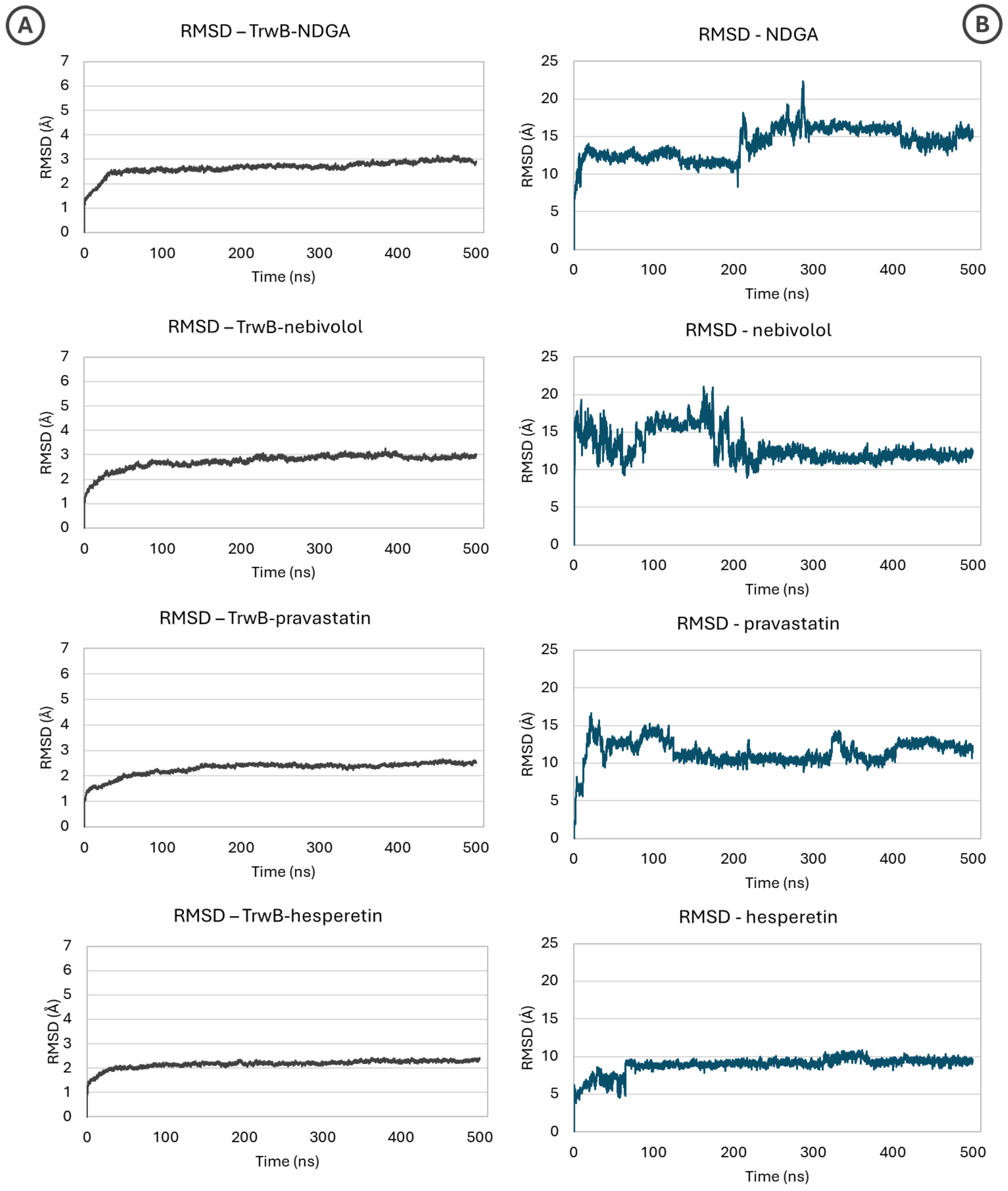

Figure S3. RMSD of the MD simulation (500 ns) of TrwB hexamer in complex with NDGA, nebivolol, pravastatin and hesperetin. A) RMSD of the protein (Cα); B) RMSD of the ligand (heavy atoms).

Pockets identified with SiteMap in the TrwB monomer.

The first pocket (site 1, Figure 8) consisted of residues G133, R141, P159, N160, N312 I315, W317, R318, E319, D356, S360 and Q386. The second pocket (site 2) is enclosed by residues L126, L127, V128, N129, G130, G384, L385, Q386, S387, T388, S389, L391, D392, V397, Q401, R404, A405, F407, R408, S409, L410, V411, V412, L413, G414, G415, D419, K421, T422, N423, D425, M426, S429, L430, G431, R460, V462, P464, I467, V477, G478, F479, A480, G481, N482, R483, P484, I485. The third pocket (site 3) corresponded to residues R119, G147, L148, R150, G151, D152, R153, R169, K171, S303, W304, L305, E306, D307, P308, N309, G310, G311, N312, L313, L343, K348, R349, R350, L351, W352, R380. The fourth (site 4) pocket and the fifth pocket (site 5) are contiguous, and they include residues P206, R207, K209, D211, A213, D286, K287, R326, K363 and D393, and R207, K209, D211, K363, L364, A365, A368, D369, K398, E399, T402 and S404, respectively (Figure 8). Two of the pockets, 1 and 3, were discarded for matching the nucleotide binding domain and for being away from the oligomerization interface, respectively.

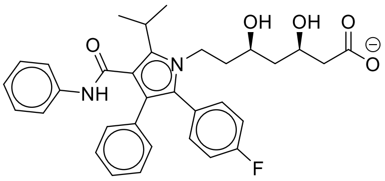

ZINC3920719
Atorvastatin

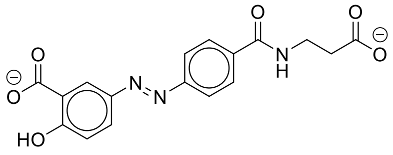

ZINC3952881
Balsalazide

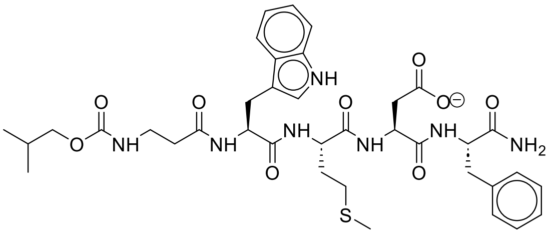

ZINC95617677
Pentagastrin

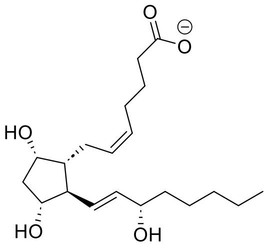

ZINC3830709
Dinoprost

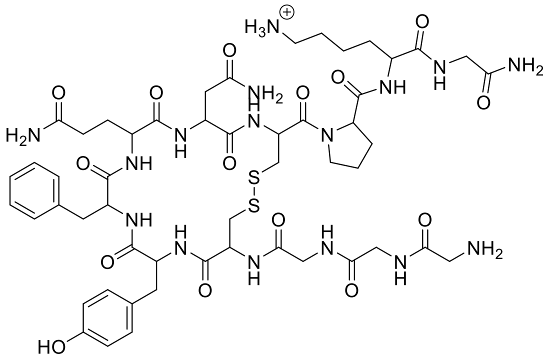

DB02638
Terlipressin

Figure S4. Chemical structure of selected compounds from the virtual screening in the monomer. The protonation states are depicted as they were employed for the VS and the MD simulations.

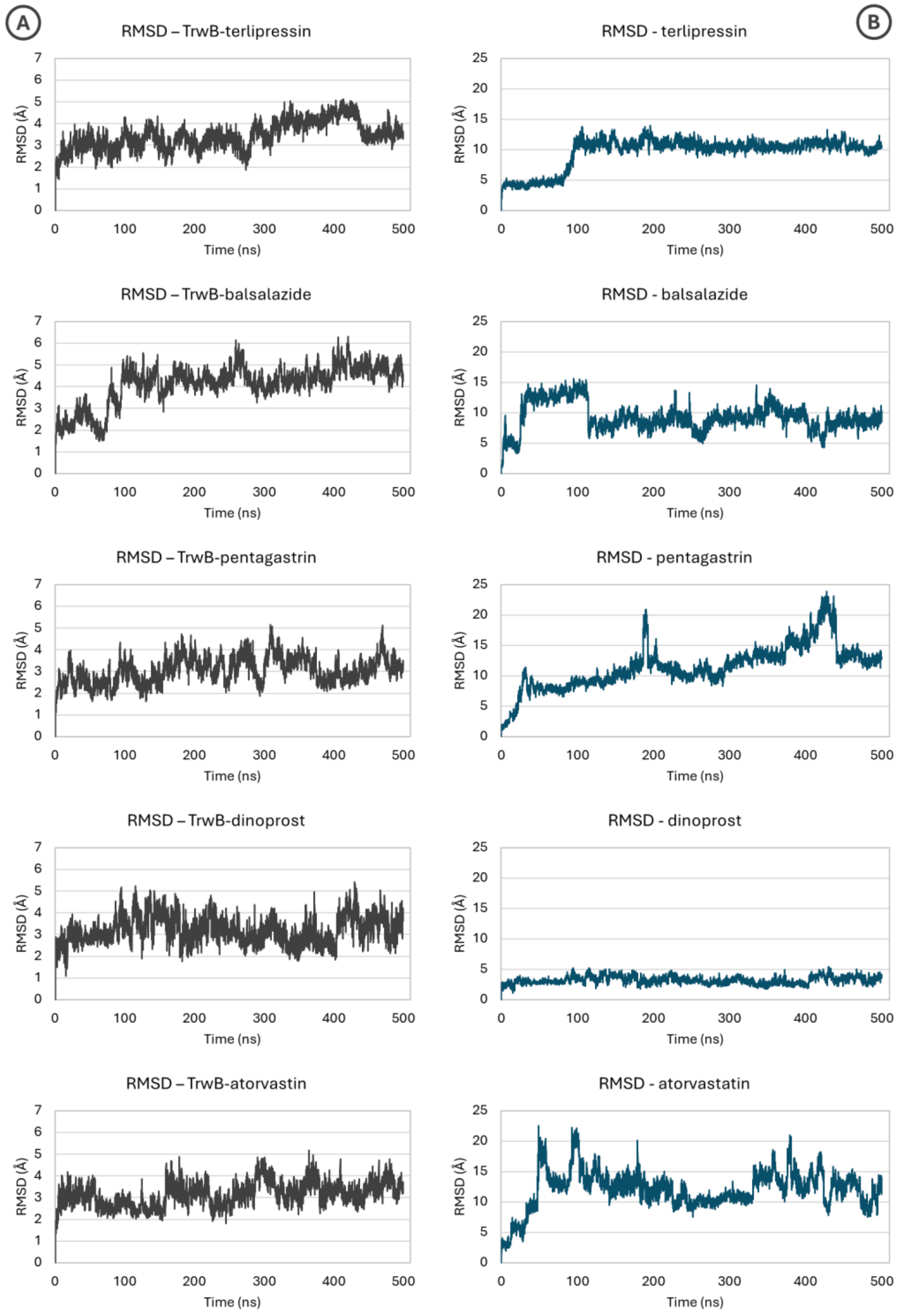

Figure S5. RMSD of the MD simulation (500 ns) of TrwB monomer in complex with terlipressin, balsalazide, pentagastrin, dinoprost and atorvastatin. A) RMSD of the protein (Cα); B) RMSD of the ligand (heavy atoms).

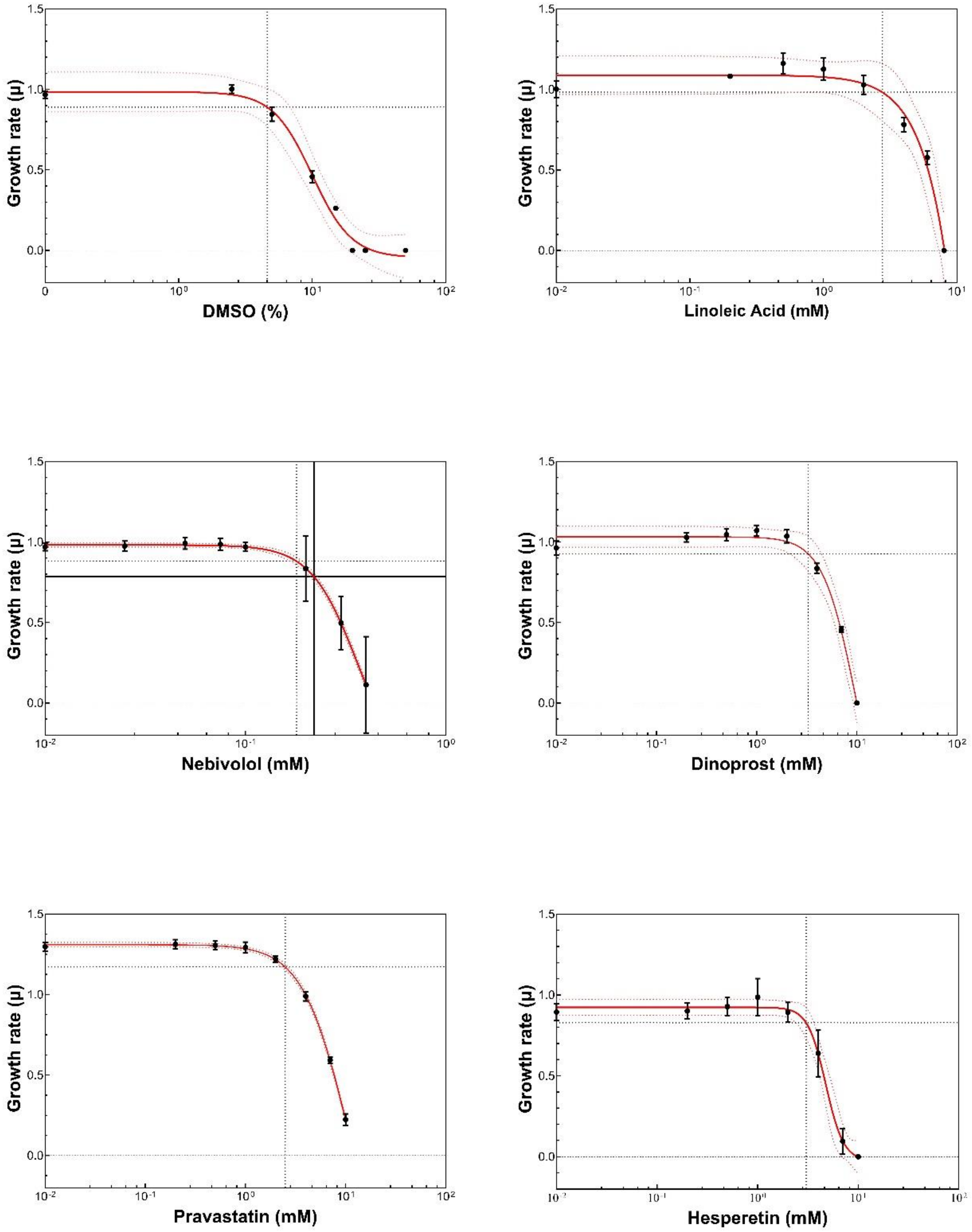

Figure S6. Effect of DMSO, linoleic acid, nebivolol, pravastatin, hesperetin, and dinoprost on donor bacterial growth. *E. coli* DH5α strain that contains R388 conjugative plasmid was incubated in the presence of different concentrations of DMSO, linoleic acid, nebivolol, pravastatin, hesperetin, or dinoprost. Cell growth was monitored by recording OD_{600} every 10 minutes during 48 h to obtain cell growth curves for donor strains. The growth rate (μ) values for each concentration of each compound were determined based on the logarithmic change in bacterial population over time. Calculations were performed using the QurvE (Quantitative Growth Curve Evaluation) R package.

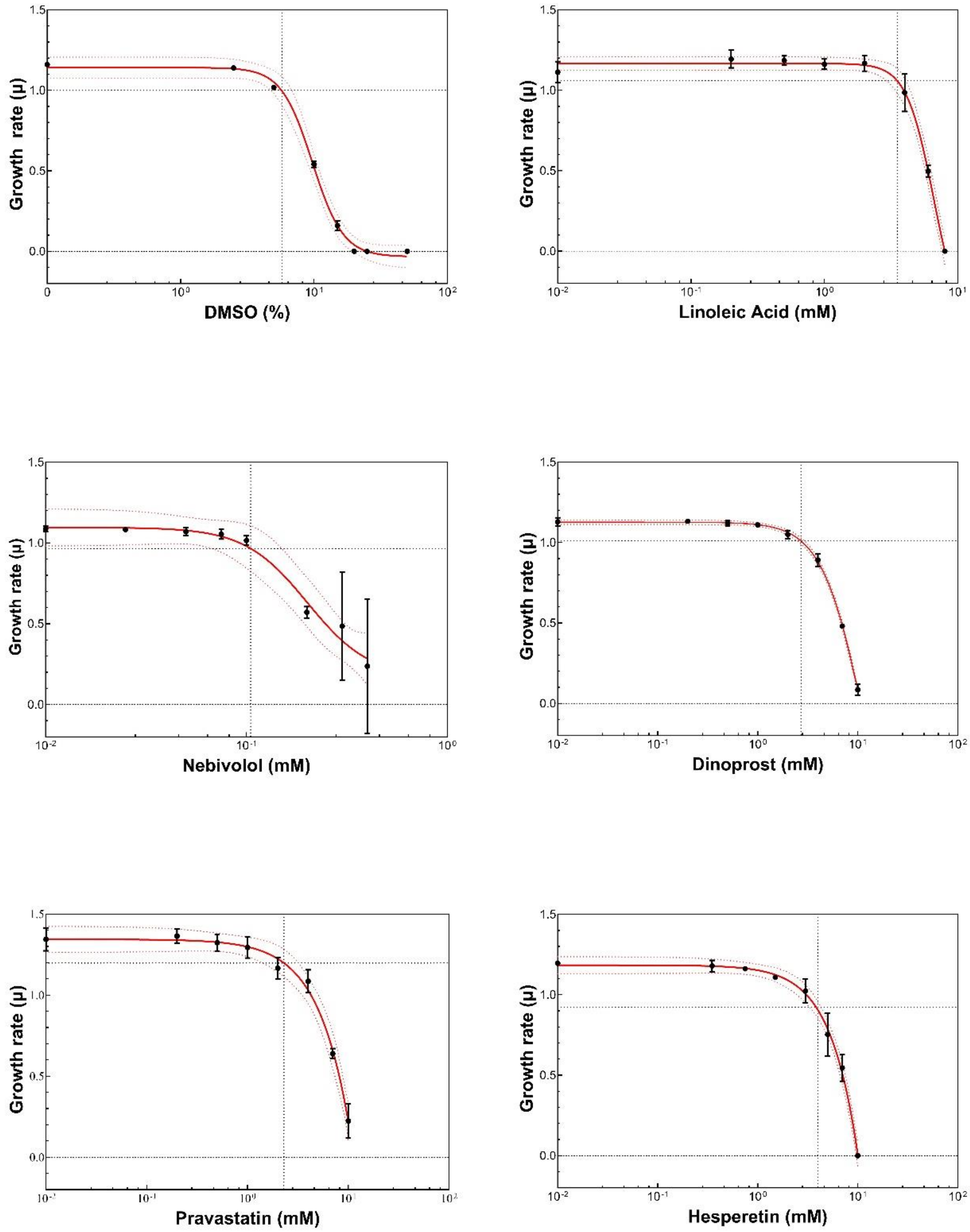

Figure S7. Effect of DMSO, linoleic acid, nebivolol, pravastatin, hesperetin, and dinoprost on recipient bacterial growth. *E. coli* HMS174 strain was incubated in the presence of different concentrations of DMSO, linoleic acid, nebivolol, pravastatin, hesperetin, or dinoprost. Cell growth was monitored by recording OD_{600} every 10 minutes during 48 h to obtain cell growth curves for donor strains. The growth rate (µ) values for each concentration of each compound were determined based on the logarithmic change in bacterial population over time. Calculations were performed using the QurvE (Quantitative Growth Curve Evaluation) R package.

Table S1. Maximum concentrations of each compound used in the bacterial conjugation inhibition experiments, determined as the highest common concentration that inhibited less than 10% growth of both donor and recipient strains.

Compound	Donor	Recipient	Maximum concentration to be used in bacterial conjugation inhibition
Linoleic acid (mM)	2.88	3.48	0.2
Nebivolol (mM)	0.18	0.106	0.075
Pravastatin (mM)	2.5	2.2	1.5
Hesperetin (mM)	3	2.38	2.0
Dinoprost (mM)	3.25	2.66	2.0
